# Metabolic Profiling of Rhizobacteria *Serratia plymuthica* and *Bacillus subtilis* Revealed Intra- and Interspecific Differences and Elicitation of Plipastatins and Short Peptides Due to Co-cultivation

**DOI:** 10.3389/fmicb.2021.685224

**Published:** 2021-05-31

**Authors:** Riya C. Menezes, Birgit Piechulla, Dörte Warber, Aleš Svatoš, Marco Kai

**Affiliations:** ^1^Research Group Mass Spectrometry/Proteomics, Max-Planck Institute for Chemical Ecology, Jena, Germany; ^2^Department of Biochemistry, University of Rostock, Institute for Biological Sciences, Rostock, Germany

**Keywords:** microbial interaction, metabolic profiling, *Serratia plymuthica*, *Bacillus subtilis*, sirius, global natural products social molecular networking, plipastatins

## Abstract

Rhizobacteria live in diverse and dynamic communities having a high impact on plant growth and development. Due to the complexity of the microbial communities and the difficult accessibility of the rhizosphere, investigations of interactive processes within this bacterial network are challenging. In order to better understand causal relationships between individual members of the microbial community of plants, we started to investigate the inter- and intraspecific interaction potential of three rhizobacteria, the *S. plymuthica* isolates 4Rx13 and AS9 and *B. subtilis* B2g, using high resolution mass spectrometry based metabolic profiling of structured, low-diversity model communities. We found that by metabolic profiling we are able to detect metabolite changes during cultivation of all three isolates. The metabolic profile of *S. plymuthica* 4Rx13 differs interspecifically to *B. subtilis* B2g and surprisingly intraspecifically to *S. plymuthica* AS9. Thereby, the release of different secondary metabolites represents one contributing factor of inter- and intraspecific variations in metabolite profiles. Interspecific co-cultivation of *S. plymuthica* 4Rx13 and *B. subtilis* B2g showed consistently distinct metabolic profiles compared to mono-cultivated species. Thereby, putative known and new variants of the plipastatin family are increased in the co-cultivation of *S. plymuthica* 4Rx13 and *B. subtilis* B2g. Interestingly, intraspecific co-cultivation of *S. plymuthica* 4Rx13 and *S. plymuthica* AS9 revealed a distinct interaction zone and showed distinct metabolic profiles compared to mono-cultures. Thereby, several putative short proline-containing peptides are increased in co-cultivation of *S. plymuthica* 4Rx13 with *S. plymuthica* AS9 compared to mono-cultivated strains. Our results demonstrate that the release of metabolites by rhizobacteria alters due to growth and induced by social interactions between single members of the microbial community. These results form a basis to elucidate the functional role of such interaction-triggered compounds in establishment and maintenance of microbial communities and can be applied under natural and more realistic conditions, since rhizobacteria also interact with the plant itself and many other members of plant and soil microbiota.

## Introduction

In nature, bacteria live in diverse and dynamic communities (Fierer and Jackson, 2006; Little et al., 2008; Phelan et al., 2012). One hotspot of bacterial life is the rhizosphere, which represents the area of soil that surrounds plant roots (Hiltner, 1904; Reinhold-Hurek et al., 2015). Plants release their excess photosynthetic metabolites including organic acids, amino acids, sugars, peptides or proteins via the root into the rhizosphere (Vančura, 1964; Smith, 1969). As consequence of this accumulation of nutrients, bacteria flourish in the soil closely associated to the plant roots (Badri and Vivanco, 2009).

Bacteria living in the rhizosphere are termed as rhizobacteria. Together with surrounding neighboring bacterial cells they form diverse microbial communities (Shank, 2018). The microbial community itself has a high impact on plant growth and development (Lindow and Brandl, 2003; Buee et al., 2009; Berendsen et al., 2012; Vorholt, 2012). On one hand, community members beneficially influence plant growth by fixation of nitrogen, release of plant hormones, defending against plant pathogens or emission of volatiles (Ryu et al., 2003; Gray and Smith, 2005; Kai et al., 2007). On the other hand, some members of the microbial community can evoke plant diseases by secretion of cell wall degrading enzymes and toxins (Van der Wolf and De Boer, 2014). Thus, the composition of the microbiome is important as it influences plants in multiple ways. The structure of microbial communities has been ascertained for roots of several plant species, however, how interaction between members of the plant-associated microbiota influence the establishment of the microbial community is not fully understood (Hassani et al., 2018). Beside plant primary and secondary metabolites, the excreted metabolites released by different members of the microbial community are considered as primary drivers of microbial community interactions (Traxler and Kolter, 2015; Shank, 2018). Individual bacteria, for instance, outcompete bacterial rivals by toxin, antibiotic and siderophore production, sense their environment by secreting signaling compounds or develop cooperative partnerships by exchanging metabolites (Griffin et al., 2004; Waters and Bassler, 2005; Phelan et al., 2012; Pande et al., 2015; Lemfack et al., 2016).

Due to the complexity of the microbial communities and the difficulties in accessing the rhizosphere, investigations of interactive processes within this bacterial network are challenging (Bonkowski, 2019). Synthetic and reductionistic approaches can help to understand underlying principles of interactions in microbial communities (Ghoul and Mitri, 2016; Røder et al., 2016; Shank, 2018). Therefore, in order to better understand causal relationships between members of the microbial community of plants, we started to investigate the interaction potential of rhizobacteria using structured, low-diversity model communities. Limitation of such *in vitro* co-cultivation approaches is to capture the actual metabolic profile theoretically involved under natural and more realistic conditions, since rhizobacteria interact with the plant itself and many other members of plant and soil microbiota. However, the main benefit of low-diversity, pair-wise bacterial colonies is the clear assignment of causative and responding strain, which can hardly be shown with the use of highly complex communities. Using this approach, we showed that co-cultivation of two rhizobacteria *Serratia plymuthica* 4Rx13 and *Bacillus subtilis* B2g revealed a distinct and wide interaction zone (Kai and Piechulla, 2018) indicating a high potential of interspecies interaction between both partners. In the present study, we questioned how metabolic features reflect this interaction. Since also closely related species inhabit same habitats, we were further interested in the intraspecific interaction potential of *S. plymuthica* 4Rx13 and chose the rhizobacterium *S. plymuthica* AS9 as model partner.

In order to get a comprehensive overview of the interplay, we extracted the metabolites released over 4 weeks of co-cultivation at specific time points and compared these metabolite profiles with the excreted metabolite profiles of mono-cultivated strains using multivariate data analyses. Furthermore, we started with the evaluation of most prominent co-cultivation correlating patterns using classical, manual identification of high resolution (HR) mass spectra (MS^1^ and MS/MS) and computational HRMS/MS identification using Global Natural Product Molecular Networking (GNPS, Wang et al., 2016) and Sirius (Dührkop et al., 2019). Our results demonstrate that the release of metabolites by rhizobacteria alters due to growth and induced by social interactions between single members of the microbial community.

## Materials and Methods

### Bacterial Cultures

*Bacillus subtilis* B2g, *Serratia plymuthica* 4Rx13 (formally known as *S. odorifera* 4Rx13) (Weise et al., 2014) and *Serratia plymutica* AS9 were originally isolated from *Brassica napus* (Marten et al., 2000; Alström, 2001; Berg et al., 2002; Neupane et al., 2012). All bacterial isolates were cultivated on nutrient agar II short-term cultures (NAII; peptone from casein 3.5 gl^−1^, peptone from meat 2.5 gl^−1^, peptone from gelatin 2.5 gl^−1^, yeast extract 1.5 gl^−1^, NaCl 5 gl^−1^, agar-agar 15 gl^−1^, pH 7.2, maximum 3 weeks old) or in liquid nutrient broth II (NBII: NAII without agar) at 30°C.

### Bacterial Self-paired and Co-cultures

One colony was picked from a short-term culture and inoculated in 6 ml of NBII-medium. After 24 h of inoculation at 160 rpm (Bühler, Tübingen, Germany) and 30°C, the cultures were diluted with NBII to obtain a starting OD_600_ of 0.05. Twenty microliters of this diluted culture were dropped in overlaps on NBII containing agar (two droplets—one droplet per strain). Self-paired overlapping droplets were used as control mono-cultivations. Co-cultivation of strains were performed for 30 days at 20°C. Investigations were conducted in two independent setups with each three replicates from three different pre-cultures for *S. plymuthica* 4Rx13 and *B. subtilis* B2g interaction and one setup with each three replicates from three different pre-cultures for *S. plymuthica* 4Rx13 and *S. plymuthica* AS9 interaction.

### Determination of Bacterial Growth During Co-cultivation

Cell growth was monitored by determination of colony forming units (cfu) within 30 days of growth (day 1, 3, 6, 7, 10, 14, 21, 28, 30). Both strains together were scraped from the agar with a pipette tip. The scraped cells were transferred into an Eppendorf tube filled with 3 ml NaCl solution (0.85%) and vortexed for 2 min. From this suspension a serial dilution was prepared with NaCl solution (0.85%) to obtain inocula of maximum 200 cells/10 μl. Droplets (each of 10 μl) of the last dilution (10^−3^-10^−8^ depending on the growth) were spotted onto a NAII agar containing Petri dish and spread to form thin lines. These Petri dishes were incubated at 30°C for 24 h to count the cells.

### Metabolite Extraction

Metabolites were extracted from the agar medium according to Tyc et al. (2017) within 30 days of growth (day 1, 3, 6, 7, 10, 14, 21, 28, 30). After harvesting the cells from the agar, the agar was cut into slices of 13.6 cm^2^ (4 × 3.4 cm). As control, the agar from non-inoculated agar was sliced into pieces of same size. These slices were transferred into Falcon tubes (50 ml, Carl Roth, Karlsruhe, Germany), which were immediately frozen in liquid nitrogen. The sample containing Falcon tubes were subsequently lyophilized for 72 h (Alpha 1-4 LSCbasic, Christ, Osterode am Harz, Germany). After lyophilization samples were crushed in a ceramic mortar containing 6 ml liquid nitrogen using a pestle. The powder was transferred into an Eppendorf tube (1.5 ml) using a paper funnel. Subsequently, one ml of 75% methanol including 10 μM indole-3-propionic acid (internal standard) was added to 125 mg powder. This mixture was vortexed for 30 s and subsequently sonicated for 30 min in a chilled water-bath at 6°C (after 15 min tubes were shortly shaken). The tubes were afterwards vortexed for 10 s and centrifuged at 2,900 g for 10 min at 4°C. The supernatants were transferred into other 1.5 ml Eppendorf tubes, which were centrifuged at 2,900 g for 15 min at 4°C. The supernatants were again transferred into new Eppendorf tubes (1.5 ml), which were stored at −70°C. At the end of this approach a sample set of each independent setup was non-stop analyzed using UHPLC/ESI-Q Exactive HF-X-MS/MS.

### UHPLC-ESI-Q Exactive MS/MS Analysis of Extracted Metabolites

Ultra-high performance liquid chromatography-tandem mass spectrometry (UHPLC/ESI-Q Exactive HF-X-MS/MS) analyses of the bacterial extracts were performed on a QE-HF-X equipped with an Ultimate 3000 series RSLC (Dionex, Sunnyvale, CA, USA) LC. Chromatographic separation was achieved on an Acclaim C18 column (150 × 2.1 mm, 2.2 μm particles with 120 Å pore diameter, Dionex, Sunnyvale, CA, USA) with a flow rate of 300 μl min^−1^ in a binary solvent system of water (solvent A) and acetonitrile (solvent B), both containing 0.1% (v/v) formic acid. Five μl each of extract was loaded onto the column and eluted by using a gradient as follows: linear increase from 0% B to 100% B within 15 min−100% B constant for 5 min—equilibration time at 0% B for 5 min. The mass spectrometer was operated in positive ionization mode using Heated-Electrospray Ionization (H-ESI). The source parameters were set to 4 kV for spray voltage, 35 V for transfer capillary voltage, capillary temperature 300°C and Funnel RF of 40 V. Fragmentation was performed using data-dependent acquisition mode with MS^1^ full scan at *m/z* 150–1,500 at 60,000 m/Δm resolving power and up to five MS/MS scans (TOP5) of the most abundant ions per duty cycle with 30,000 m/Δm resolving power and stepped normalized collision energy of 20, 30 and 40. Data was evaluated and interpreted using Xcalibur v.3.0.63 software (Thermo Fisher Scientific, Waltham, MA, USA).

### Data Processing

Conversion of raw data files to mzXML format files was performed using MSConvertGUI tool of the ProteoWizard 3.0.X software (Chambers et al., 2012). Further reprocessing was conducted either via uploading the mzXML files to XCMS Online (Tautenhahn et al., 2012) or manually via the XCMS package in RStudio version 1.0.153 (Smith et al., 2006; Tautenhahn et al., 2008; Benton et al., 2010).

For manual XCMS processing, feature detection was performed using *centWave* algorithm with following parameter: Δ *m/z* 5 ppm, peak width from 5 to 20 s [Command in RStudio CentWaveParam (ppm = 5, peakwidth = c(5, 20), integrate = 1, fitgauss = TRUE, snthresh = 30, mzdiff = −0.001)]. Retention time correction was performed using Obiwarp method (Prince and Marcotte, 2006). Chromatograms were aligned with minfrac = 0 and bw = 0.5.

For XCMS Online processing multigroup analysis in version 3.7.1 was applied. Feature detection was performed using *centWave* algorithm with following parameter: Δ *m/z* 5 ppm, peak width from 5 to 20 s, Signal/Noise threshold = 4, mzdiff = 0.01, integration method = 1, prefilter peaks 3, prefilter intensity = 100 and noise filter = 100. Retention time correction was performed using Obiwarp method (profStep = 1). Parameter for alignment were mzwid = 0.025, bw = 0.5, minfrac = 0.5, minsamp = 1, max = 100.

### Data Analysis

Partitioning around medoids (PAM) clustering (Kaufman and Rousseeuw, 1990) was performed from the manually processed XCMS data in RStudio. Principal component analyses (PCAs) were performed using XCMS Online data. Further data processing, statistical analysis including correlation pattern analysis using Pearson r as distance measure was performed using Metaboanalyst 5.0 (ref. for preliminary versions, Xia et al., 2009; Chong et al., 2019; Pang et al., 2020). In MetaboAnalyst 5.0 the tool “Statistical analysis” was applied. After import, data were filtered using interquantile range and normalized using auto scaling. All analyses were performed two times (technical replicates) for every condition with three biological replicates each (except when indicated).

### Identification

#### Classical, Manual Identification of High Resolution Mass Spectra

Classical identification was performed using accurate mass measurements of mass features (MS^1^ and MS/MS). A mass tolerance of ±3 ppm was applied as threshold between accurate and exact mass. For plipastatin isomer identification, several diagnostic marker ions were used accordingly for the amino acids at position 6 and 10 of the plipastatin molecule (Pathak et al., 2012; Kaki et al., 2020). For plipastatin A1, we used *m/z* 966.45672 (corresponding to chemical formula C_46_H_64_N_9_O_14_) and *m/z* 1080.53603 (corresponding to chemical formula C_51_H_74_N_11_O_15_) representing alanine^6^/isoleucine^10^. For plipastatin A2 we used *m/z* 1066.52038 (corresponding to chemical formula C_50_H_72_N_11_O_15_) representing alanine^6^/valine^10^. For plipastatin B1 we used *m/z* 994.48802 (corresponding to chemical formula C_48_H_68_N_9_O_14_) and *m/z* 1108.56733 (corresponding to chemical formula C_53_H_78_N_11_O_15_) representing valine^6^/isoleucine^10^. For plipastatin B2 we used *m/z* 980.47375 (corresponding to chemical formula C_47_H_66_N_9_O_14_) and *m/z* 1094.55078 (corresponding to chemical formula C_52_H_76_N_11_O_15_) representing valine^6^/valine^10^.

#### Determination of Chemical Formulae Using Sirius 4

Conversion of raw data files to mzML format files was performed using MSConvertGUI tool of the ProteoWizard 3.0.X software (Chambers et al., 2012). The mzML files were imported into MZmine 2.53 (Pluskal et al., 2010) and processed as follows. MS^1^ and MS/MS level detection with a noise level of 1.0E3. Chromatogram builder (MS^1^ level) with a minimal time span of 0.01 min, a minimal height of 3.0E3 and a mass tolerance (*m/z*) of 5 ppm. Deisotoping was performed with a mass tolerance (*m/z*) of 5 ppm and a retention time tolerance of 0.2 min from ions with maximum charges of 2. Data were exported as mgf files and subsequently imported into Sirius 4.5.3 (Dührkop et al., 2019). For identification we considered the Sirius score, the fragmentation tree score (Rasche et al., 2012; Böcker and Dührkop, 2016) and isotopic pattern analysis (Böcker et al., 2009). For Oocydin A assignment, we further used CSI:FingerID (Dührkop et al., 2015).

#### Molecular Networking Workflow

A molecular network was created according to Wang et al. (2016) using the online workflow (https://ccms-ucsd.github.io/GNPSDocumentation/) on the GNPS website (http://gnps.ucsd.edu). The data was filtered by removing all MS/MS fragment ions within +/– 17 Da of the precursor *m/z*. MS/MS spectra were window filtered by choosing only the top 6 fragment ions in the +/– 50Da window throughout the spectrum. The precursor ion mass tolerance was set to 2.0 Da and a MS/MS fragment ion tolerance of 0.5 Da. A network was then created where edges were filtered to have a cosine score above 0.7 and more than 6 matched peaks. Further, edges between two nodes were kept in the network if and only if each of the nodes appeared in each other's respective top 10 most similar nodes. Finally, the maximum size of a molecular family was set to 100, and the lowest scoring edges were removed from molecular families until the molecular family size was below this threshold. The spectra in the network were then searched against GNPS' spectral libraries. The library spectra were filtered in the same manner as the input data. All matches kept between network spectra and library spectra were required to have a score above 0.7 and at least 6 matched peaks. The IDs of the respective jobs that were used for this study are ID = 6b27a2bb84ff491bb9ef8b7fb5cea822 for *S. plymuthica* 4Rx13 and AS9 interaction and ID = 0bb4c822bc4141f09a4ed723152db975 for *S. plymuthica* 4Rx13 and *B. subtilis* B2g interaction.

## Results

### Metabolic Profiles From Mono-cultivated Isolates *S. plymuthica* 4Rx13 and AS9 and *B. subtilis* B2g Differ During Cultivation

**Figure 1:**
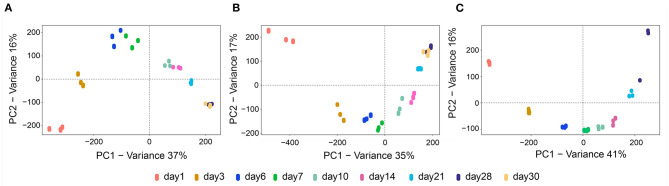
Metabolic profiles from mono-cultivated isolates *S. plymuthica* 4Rx13 and AS9 and *B. subtilis* B2g differ during cultivation. PCA plots of mono-cultivated *S. plymuthica* 4Rx13 **(A)** *B. subtilis* B2g **(B)** and *S. plymuthica* AS9 **(C)**.

Before the interaction of the rhizobacteria was investigated, we analyzed the metabolic features of the mono-cultivated isolates *S. plymuthica* 4Rx13, *S. plymuthica* AS9 and *B. subtilis* B2g during cultivation on solid medium. Since we were interested in metabolites excreted into the environment, we extracted agar slices using a recently established protocol (Tyc et al., 2017) at different time points (day 1, 3, 6, 7, 10, 14, 21, 28, and 30). The respective extracts were non-targeted analyzed using UHPLC/HRMS and processed with XCMS Online (Tautenhahn et al., 2012). To evaluate the dynamics of metabolite production during cultivation of the mono-cultivated isolates, unsupervised multivariate PCAs were conducted. These PCAs showed a clear separation of the released metabolites in relation to the time point of cultivation of all three tested bacterial isolates. Thereby, the biological replicates of each time point grouped together (Figure 1). For *S. plymuthica* 4Rx13, PC1 and PC2 explained 37% and 16% of the total variability, respectively (Figure 1A). For *B. subtilis* B2g, PC1 and PC2 explained 35 and 17% of the total variability, respectively (Figure 1B). For *S. plymuthica* AS9, PC1 and PC2 explained 41 and 16% of the total variability, respectively (Figure 1C). The metabolic profiles of all three bacteria were mainly separated along PC1, however, a cultivation dependent separation was also observed along PC2. Metabolite profiles from day 28 and day 30 did not separate neither along PC1 nor along PC2. These results clearly show a variation of metabolic profiles during the time of bacterial cultivation.

### Metabolic Profiles of *S. plymuthica* 4Rx13 Differ Interspecifically to *B. subtilis* B2g and Intraspecifically to *S. plymuthica* AS9

**Figure 2:**
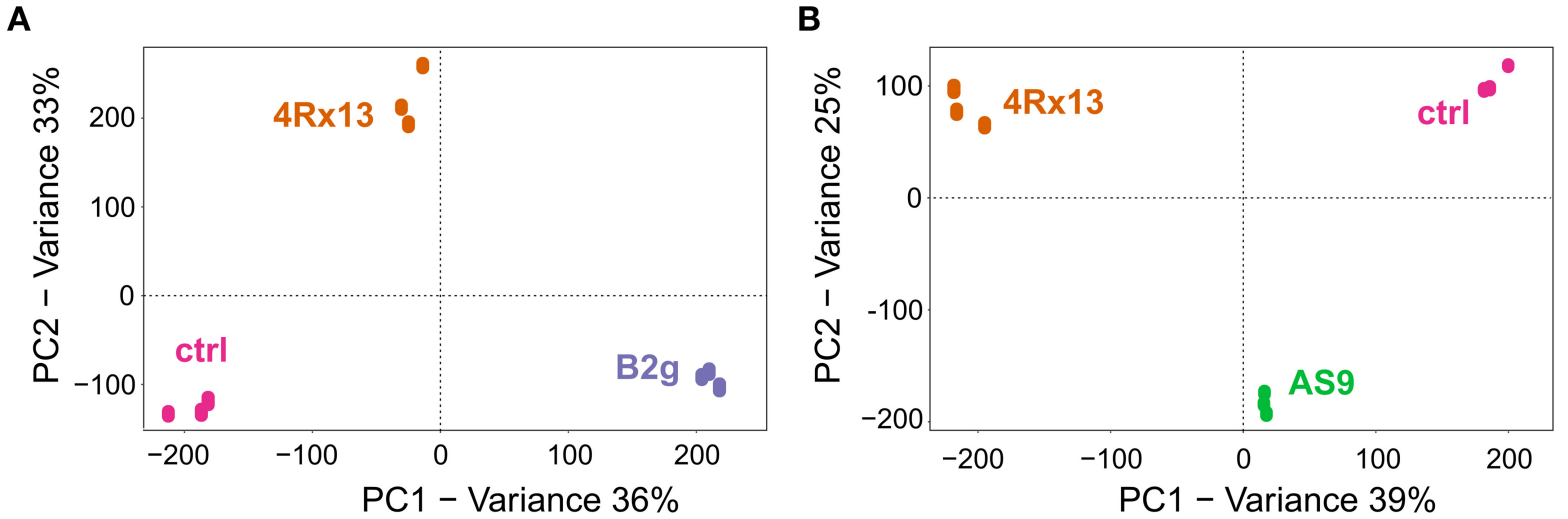
Metabolic profiles of *S. plymuthica* 4Rx13 differ interspecifically to *B. subtilis* B2g and intraspecifically to *S. plymuthica* AS9. PCA plots of mono-cultures of **(A)** *S. plymuthica* 4Rx13 and *B. subtilis* B2g and ctrl (extracted pure agar) and **(B)** *S. plymuthica* 4Rx13 and *S. plymuthica* AS9 and ctrl (control, extracted non-inoculated agar).

To evaluate whether the metabolic profiles of the three mono-cultivated bacteria can differ in relation to each other, additional PCAs of the processed data from day 6 were exemplarily conducted. The metabolic profiles of *S. plymuthica* 4Rx13 and *B. subtilis* B2g and control (extracted non-inoculated agar) clearly separated from each other (Figure 2). The biological replicates of each cultivation also grouped together (Figure 2A). PC1 and PC2 explained 36 and 33% of the total variability, respectively. Thereby, 338 features (*m/z*/RT) and 414 features (*m/z*/RT) were exclusively present or significantly increased in *S. plymuthica* 4Rx13 (correlation > 0.9, *p* < 0.001) and *B. subtilis* B2g, respectively (correlation > 0.9, *p* < 0.001).

Surprisingly, the metabolites of *S. plymuthica* 4Rx13 and AS9 also showed distinct and separated pattern (Figure 2B). PC1 and PC2 explained 39% and 25% of the total variability, respectively. Thereby, 655 features (*m/z*/RT) and 72 features (*m/z*/RT) were exclusively present or significantly increased in *S. plymuthica* 4Rx13 and *S. plymuthica* AS9, respectively (correlation > 0.9, *p* < 0.001).

These results demonstrated that the metabolic profiles of *S. plymuthica* 4Rx13 differ both interspecifically to *B. subtilis* B2g metabolites, but also intraspecifically to *S. plymuthica* AS9 metabolites.

### Release of Different Secondary Metabolites Represents a Contributing Factor to Inter- and Intraspecific Variations in Metabolite Profiles

**Figure 3:**
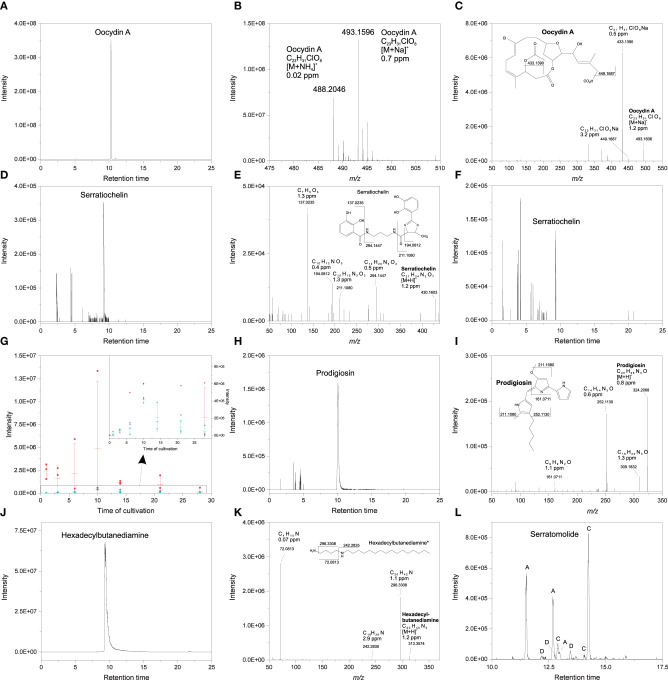
Secondary metabolites produced by *S. plymuthica* 4Rx13 at day 6 of cultivation **(A)** EIC of putative Oocydin A (*m/z* 493.15–493.17) **(B)** High resolution mass spectrum of putative Oocydin A **(C)** HCD MS/MS of putative Oocydin A **(D)** EIC of putative Serratiochelin A (*m/z* 430.159–430.168) produced by *S. plymuthica* 4Rx13 **(E)** HCD MS/MS of putative Serratiochelin **(F)** EIC of putative Serratiochelin A (*m/z* 430.159–430.168) produced by *S. plymuthica* AS9 **(G)** Abundance of putative Serratiochelin in *S. plymuthica* 4Rx13 (red) and AS9 (blue) (inserted window presents an enlarged section (black labeled box), dots are replicates, minus are medians with standard deviations) **(H)** EIC of putative Prodigiosin (*m/z* 493.15–493.17) **(I)** HCD MS/MS of putative Prodigiosin **(J)** EIC of putative hexadexylbutanediamine (*m/z* 313.35–313.36) **(K)** HCD MS/MS of putative hexadexylbutanediamine (*full structural characterization needs to be confirmed). **(L)** EIC of different putative Serratomolide homologs (A = *m/z* 515.33–515.34, C = *m/z* 543.35–543.37, D = *m/z* 529.34–529.36).

To explore whether secondary metabolites produced by *S. plymuthica* 4Rx13, *S. plymuthica* AS9 and *B. subtilis* B2g reflect inter- and intraspecific metabolic differences, we used a targeted approach by manually analyzing accurate masses, tandem mass spectra and computing Sirius and GNPS of exclusively present or significantly increased mass features at certain time points during growth of each strain. In *S. plymuthica* 4Rx13 we observed exclusive features with [M+Na]^+^ *m/z* 493.1596 and [M+NH_4_]^+^ *m/z* 488.2046 corresponding to the molecular formula of C_23_H_31_ClO_8_ (Δppm = 0.7, calcd. *m/z* [M+Na]^+^ 493.15997, Δppm 0.02 calcd. *m/z* [M+NH_4_]^+^ 488.2045) (Figures 3A,B). This molecular formula was confirmed by Sirius (first hit with a Sirius score of 92.9%, isotope score: 10.2 tree score: 19.62; peak explained 3/15; total explained 34.251%) and isotopic patterns and corresponds to the haterumalide Oocydin A. The tandem mass spectrum, which showed fragments at *m/z* 449.1687 (C_22_H_31_ClO_6_Na, Δppm 0.5) and *m/z* 433.1390 (C_21_H_27_ClO_6_Na, Δppm 3.2), is in agreement with previously reported data of Oocydin A (Figure 3C, Matilla et al., 2012). With 42.295% similarity at rank 1, the computational tandem mass spectrum analysis using CSI:FingerID further supported the assignment of this feature as Oocydin A. Furthermore, we detected a mass feature of [M+H]^+^ *m/z* 430.1603 corresponding to the molecular formula of C_21_H_23_N_3_O_7_ (Δppm = 1.2, calcd. *m/z* [M+H]^+^ 430.16088) (Figures 3D,E). Tandem mass spectral analysis showed fragments at *m/z* 294.1447 (C_14_H_20_N_3_O_4_, Δppm 0.5), *m/z* 211.1080 (C_10_H_15_N_2_O_3_, Δppm 1.3), *m/z* 194.0812 (C_10_H_12_NO_3_, Δppm 0.4) and *m/*z 137.0235 (C_7_H_5_O_3_, Δppm 0.02), which strongly indicated the production of the catecholate siderophore Serratiochelin A by *S. plymuthica* 4Rx13 (Figure 3E). In contrast to Oocydin A, Serratiochelin is also released by *S. plymuthica* AS9, however, in lower amounts compared to *S. plymuthica* 4Rx13 during cultivation period (Figures 3F,G). *S. plymuthica* AS9 showed an exclusive mass feature at *m/z* 324.2068 corresponding to molecular formula of C_20_H_25_N_3_O (Δppm = 0.8, calcd. [M+H]^+^ *m/z* 324.2070) (Figures 3H,I). This molecular formula was confirmed by Sirius as first hit with a Sirius score of 98.7%, tree score of 12.1%, 3 of 19 explained peaks and total explaining of 45.4%. The tandem mass spectral analysis showed fragments at *m/z* 309.1832 (C_19_H_23_N_3_O, Δppm 1.3), *m/z* 252.1130 (C_14_H_14_N_3_O, Δppm 0.6), *m/z* 161.0711 (C_9_H_9_N_2_O, Δppm 1.1) (Figure 3I), which is in agreement with entries in GNPS library for Prodigiosin (Spectrum ID CCMSLIB00005435440 and CCMSLIB00000072234). This collective information strongly indicates the presence of Prodigiosin. Prodigiosin was solely detected in *S. plymuthica* isolate AS9. Although *S. plymuthica* AS9 is genetically capable of producing broad-spectrum zeamine-related antibiotics (Masschelein et al., 2013), we did not detect mass features corresponding to these known zeamines (Zeamine I, II) in the extract of *S. plymuthica* AS9. During the search for the zeamines, we observed a *S. plymuthica* AS9 unique mass feature *m/z* 313.3576 corresponding to molecular formula of C_20_H_44_N_2_ (Δppm = 0.7, calcd. *m/z* [M+H]^+^ 313.35772; Figures 3J,K). This molecular formula corresponds to a hexadecylbutanediamine structure that shows similarities to the zeamine antibiotics (Figure 3J). Analysis using Sirius 4 revealed only one hit with a Sirius score of 100% corresponding to molecular formula of C_20_H_44_N_2_ (tree score: 20.58; peak explained 3/22; total explained 47.16%) Tandem mass spectral fragments at *m/z* 296.3308 (C_20_H_42_N, Δppm 1.1), *m/z* 242.2835 (C_16_H_36_N, Δppm 2.9) and *m/z* 72.0813 (C_4_H_10_N, Δppm 0.07) strongly indicated the hexadecylbutanediamine structure (Figure 3K), however, full structural characterization via synthesis and NMR has to be performed in future. Further putatively identified exclusive secondary metabolites from *S. plymuthica* AS9 were found to belong to the Serratomolide family (Figure 3I), including three homologs of Serratomolide A ([M+H]^+^ *m/z* 515.3343, Δppm 2, calcd. *m/z* [M+H]^+^ 515.3327 for molecular formula C_26_H_46_N_2_O_8_, which was the first hit in Sirius with a Sirius score of 87.6%, isotope score of 6.25, tree score of 56.7 by 9 of 27 peaks explained and a total explaining of 51.7%), two homologs of Serratomolide C ([M+H]^+^ *m/z* 543.3657, Δppm 2.2, calcd. *m/z* [M+H]^+^ 543.36399 for C_28_H_50_N_2_O_8_, which was the first hit in Sirius with a Sirius score of 36.8%, isotope score of 5.57, tree score of 41.69 by 8 of 29 peaks explained leading to a total explaining of 37.8%) and three homologs of Serratomolide D ([M+H]^+^ *m/z* 529.35034, Δppm 3, calcd. *m/z* [M+H]^+^ 529.3483 for C_27_H_48_N_2_O_8_). Due to low abundances of Serratomolide D homologs, we have not been able to acquire sufficient mass spectra to confirm their identity via Sirius. Future investigations will have to focus on full characterization of the Serratomolide homologs produced by *S. plymuthica* AS9.

**Figure 4:**
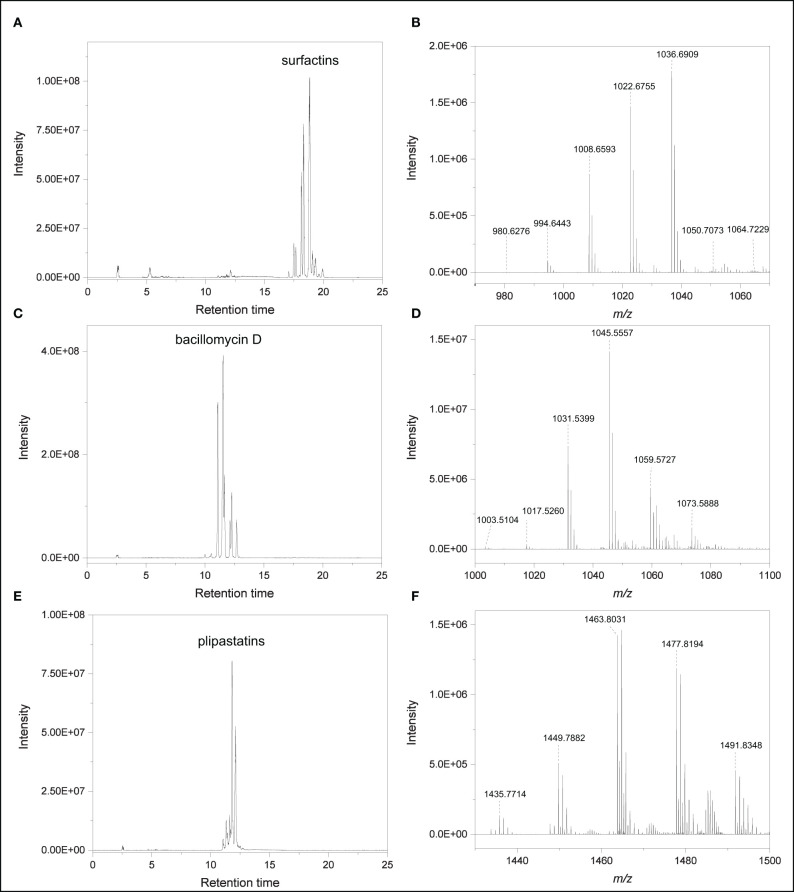
Lipopeptides produced by *B. subtilis* B2g at day 21 of cultivation **(A)** EIC of compounds putatively belonging to the surfactin family (*m/z* 982, 986, 1,010, 1,023, 1,037, 1,050, 1,051, and 1,065). **(B)** High resolution mass spectrum (RT 16.78–21.46 min). **(C)** EIC of compounds putatively belonging to the bacillomycin D family (*m/z* 1,004, 1,018, 1,046, 1,060, 1,074). **(D)** High resolution mass spectrum (RT 9.45–12.75 min). **(E)** EIC of compounds putatively belonging to plipastatin family (*m/z* 1,436, 1,450, 1,464, 1,478, 1,492). **(F)** High resolution mass spectrum (RT 9.45–12.75 min).

None of the *S. plymuthica* secondary metabolites were released by *B. subtilis* B2g. Accurate mass measurements revealed several putative lipopeptide isomers belonging to the families of surfactins, bacillomycin D and plipastatins in the extract of *B. subtilis* B2g (Figure 4, Supplementary Table 1, Table 1), which have been not observed in *S. plymuthica* isolates. Surfactins were detected at [M+H]^+^ *m/z* 980.6283 (C_49_H_85_N_7_O_13_, Δppm 0.5), [M+H]^+^ *m/z* 994.6440 (C_50_H_87_N_7_O_13_, Δppm 0.5), [M+H]^+^ *m/z* 1008.659 (C_51_H_89_N_7_O_13_, Δppm 0.1), [M+H]^+^ *m/z* 1022.6751 (C_52_H_91_N_7_O_13_, Δppm 0.4), [M+H]^+^ *m/z* 1036.6907 (C_53_H_93_N_7_O_13_, Δppm 0.3), [M+H]^+^ *m/z* 1050.7062 (C_54_H_95_N_7_O_13_, Δppm 0.2) and 1064.7222 (C_55_H_97_N_7_O_13_, Δppm 0.5) (Figures 4A,B). Members of the bacillomycin D family were observed at [M+H]^+^ *m/z* 1003.5094 (C_46_H_70_O_15_N_10_, Δppm 0.1), [M+H]^+^ *m/z* 1017.5254 (C_47_H_72_O_15_N_10_, Δppm 0.3), [M+H]^+^ *m/z* 1031.5423 (C_48_H_74_N_10_O_15_, Δppm 1.4), [M+H]^+^ *m/z* 1045.5581 (C_49_H_76_N_10_O_15_, Δppm 1.7) (Figures 4C,D). Several plipastatins were detected at [M+H]^+^ *m/z* 1449.7898 (C_71_H_108_N_12_O_20_, Δppm 1.5), [M+H]^+^ *m/z* 1463.8047 (C_72_H_110_N_12_O_20_, Δppm 1), [M+H]^+^ *m/z* 1477.8188 (C_73_H_112_N_12_O_20_, Δppm 1.4), [M+H]^+^ *m/z* 1491.8362 (C_74_H_114_N_12_O_20_, Δppm 1.1), [M+H]^+^ *m/z* 1505.84998 (C_75_H_116_N_12_O_20_, Δppm 0.1), [M+H]^+^ *m/z* 1519.8639 (C_76_H_118_N_12_O_20_, Δppm 1.2) (Figures 4E,F, Table 1). For each lipopeptide different isomers could be detected, however, complete characterization of these isomers is beyond the scope of this study.

**Table 1:** Members of the plipastatin family (known and putative variants) produced by *B. subtilis* B2g—plipastatin isomers and variants that are more pronounced in interaction are marked in red, n.d., not determined due to insufficient MS/MS.

**Name**	**Chemical formula**	**Monoisotopic mass (M) *m/z***	**Isomer**	**measured *m/z* [M+2H]^**2+**^**	**calc. *m/z* [M+2H]^**2+**^**	**Δppm**	**measured *m/z* [M+H]^**+**^**	**calcd. *m/z* [M+H]^**+**^**	**Δppm**
Plipastatin	C_71_H_108_N_12_O_20_	1448.7797	A1	725.39841	725.39742	1.4	1449.7898	1449.7875	1.5
			A1						
			A2						
Plipastatin	C_72_H_110_N_12_O_20_	1462.7954	B1	732.40649	732.40524	0.8	1463.8047	1463.8032	1
			B2						
			A1 and B2						
			A1						
Plipastatin	C_73_H_112_N_12_O_20_	1476.811	B1	739.41381	739.41307	1	1477.8208	1477.8188	1.4
			B1						
			B2						
			A1						
Plipastatin	C_74_H_114_N_12_O_20_	1490.8266	B1	746.42243	746.42089	2.1	1491.8362	1491.8387	1.1
			B2						
			A1						
Plipastatin	C_75_H_116_N_12_O_20_	1504.8423	n.d.	753.4287	753.4287	0.001	1505.84998	1505.8501	0.1
Plipastatin	C_76_H_118_N_12_O_20_	1518.86799	n.d.	760.4363	760.4365	0.3	1519.8639	1519.86581	1.2
Plipastatin V1	C_71_H_110_N_12_O_21_	1466.7902	n.d.	734.40378	734.40270	1.5	1467.7999	1467.79812	1.2
Plipastatin V2	C_72_H_112_N_12_O_21_	1480.8059496	n.d.	741.41101	741.41053	0.7	1481.8151	1482.82160	0.9
Plipastatin V3	C_73_H_114_N_12_O_21_	1494.8215996	n.d.	748.41870	748.41835	0.5	1495.83191	1495.8294246	1.7
Plipastatin V4	C_74_H_116_N_12_O_21_	1508.8372497	n.d.	755.42680	755.42618	0.8	1509.8431	1509.8451	1.3
Plipastatin V5	C_75_H_118_N_12_O_21_	1522.8523513	n.d.	762.43459	762.43400	0.7	1523.8575	1523.8607	2.1

These results show that the release of different secondary metabolites is contributing to inter- and intraspecific variations in metabolite profiles between the *S plymuthica* isolates 4Rx13 and AS9 and *B. subtilis* B2g. However, a high number of further varying, contributing factors still need to be elucidated.

### Interspecific Co-cultivation of *S. plymuthica* 4Rx13 and *B. subtilis* B2g Showed Consistently Distinct Metabolic Profiles Compared to Mono-cultivated Species

In order to evaluate whether bacterial interaction-induced changes in released metabolites can be monitored by metabolic profiling, we used a structured low diversity co-culture model between *S. plymuthica* 4Rx13 and *B. subtilis* B2g, which previously indicated an enormous potential of interaction between both partners (Kai and Piechulla, 2018).

**Figure 5:**
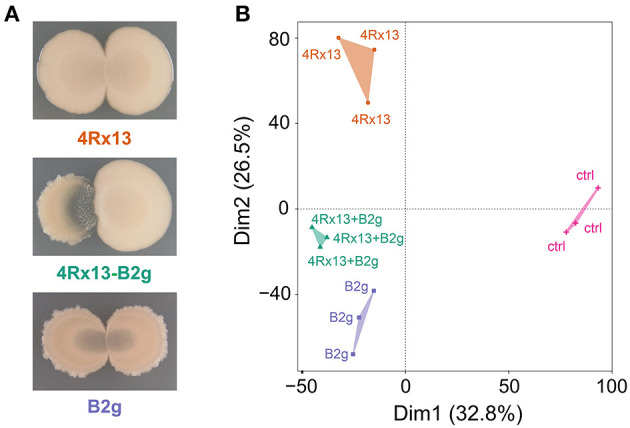
Interspecific interaction of *S. plymuthica* 4Rx13 and *B. subtilis* B2g on day 6 of cultivation. **(A)** representative photographs, **(B)** PAM-Cluster plot (computation of 4 clusters); 4Rx13 correspond to mono-cultivation of *S. plymuthica* 4Rx13, B2g correspond to mono-cultivation of *B. subtilis* B2g, 4Rx13 + B2g correspond to co-cultivation of *S. plymuthica* 4Rx13 with *B. subtilis* B2g, ctrl (control, extracted non-inoculated agar).

We reproduced the co-cultivation assays and observed that the interplay of *S. plymuthica* 4Rx13 with *B. subtilis* B2g caused a distinct and bright interaction zone [as already described in Kai and Piechulla (2018)], which was not observed in self-paired cultures (Figure 5A).

In order to evaluate how these different phenotypes are reflected by metabolic differences, we scraped off the cells of self-paired and co-cultivated bacteria from the agar and extracted the metabolites released into the agar at different time points (day 1, 3, 6, 7, 10, 14, 21, 28, and 30). These extracts were analyzed in a non-targeted approach using UHPLC/HRMS and processed with XCMS. To evaluate the metabolic profiles, PAM cluster analyses were conducted (Kaufman and Rousseeuw, 1990). PAM clustering plots from cultures at day 6 of cultivation showed four distinct clusters [Figure 5B, cluster 1–4Rx13 mono, cluster 2–4Rx13+B2g interaction, cluster 3 – B2g mono, cluster 4 – ctrl (agar)]. This clear separation was not observable at day 1 and 3 of cultivation (Supplementary Figure 1). From day 6, the clustering continued until the later stages of growth (Supplementary Figure 1, with the exemption of day 28). These results suggest that the metabolic profiles of the co-cultures of *S. plymuthica* 4Rx13 with *B. subtilis* B2g differ from the metabolic profiles of each mono-culture.

### Intraspecific Interaction of *S. plymuthica* 4Rx13 and *B. subtilis* B2g Showed Altered Mass Features Compared to Mono-cultivated Species

**Figure 6:**
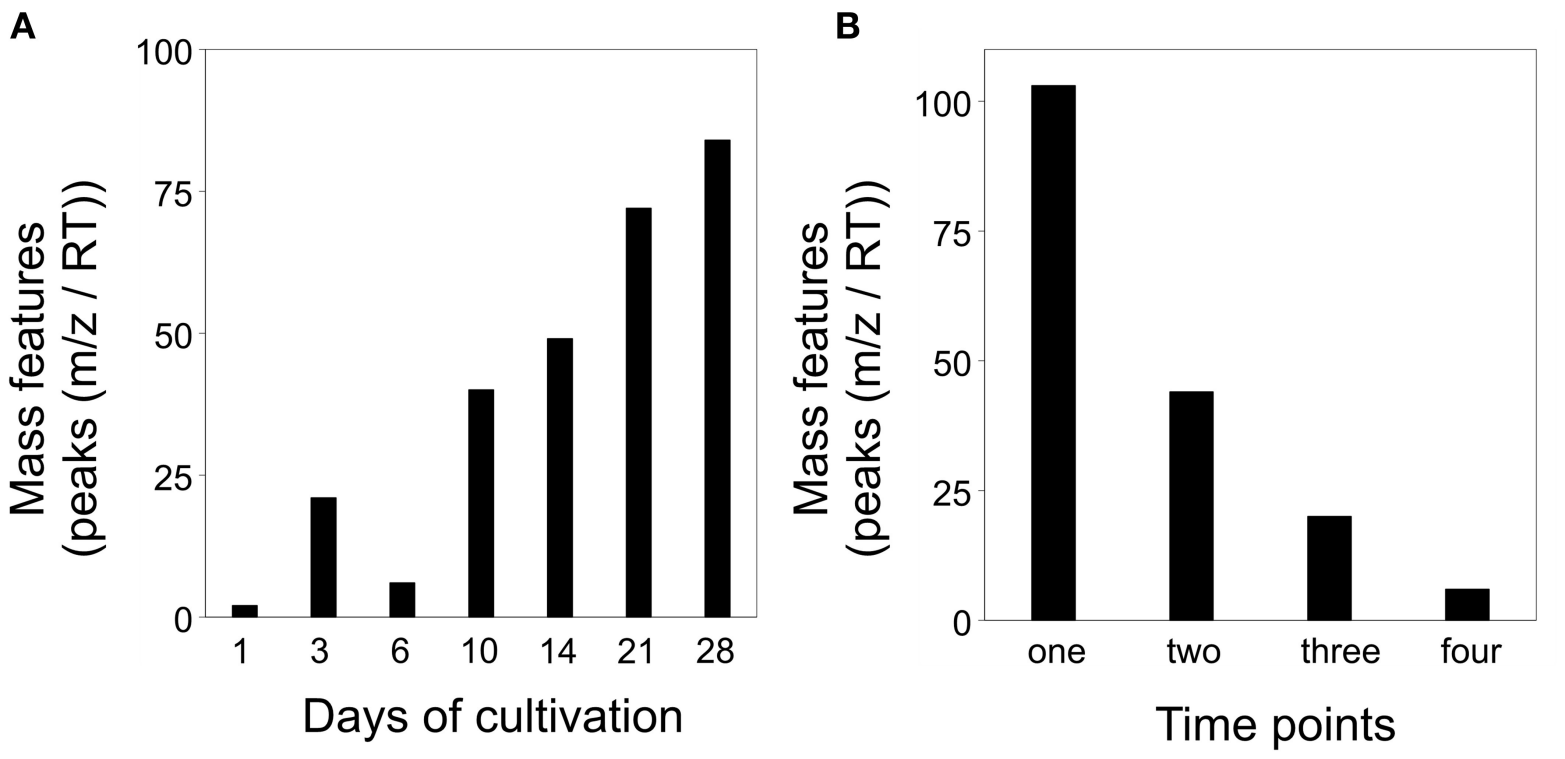
Altered mass features [peaks (*m/z*/RT)] in co-cultivation of *S. plymuthica* 4Rx13 and *B. subtilis* B2g in comparison to mono-cultivated strains and medium control (correlation threshold > 0.6) **(A)** Number of co-cultivation correlating mass features in relation to each day of cultivation (extracts were sampled at day 1, 3, 6, 10, 14, 21, and 28 of mono and co-cultivation of *S. plymuthica* 4Rx13 and *B. subtilis* B2g). **(B)** Frequency of occurrence of each co-cultivation correlating mass feature.

In order to ascertain the metabolic patterns connected to the distinct metabolic profiles, we performed correlation pattern analysis using MetaboAnalyst. Thereby, we searched for metabolic features that were altered in co-cultivation of interacting *S. plymuthica* 4Rx13 with *B. subtilis* B2g in comparison to both mono-cultivated strains and control. Setting a correlation threshold of 0.6, we found a total of 173 mass features (Supplementary Tables 2, 3). Using this approach, no mass feature was solely detected in co-cultivation. Mass features were instead differentially released by the co-cultivated strains in relation to the mono-cultivated ones (Figure 6, Supplementary Table 2). The longer both strains were co-cultivated, the more mass features were detected above the correlation threshold of 0.6 (Figure 6A). Next, we evaluated whether mass features were only altered at one single time point or over a certain time period. No mass feature was co-cultivation-induced over the whole time range. One hundred and three features (60 %) were differing at one single time point only, while 70 features (40%) were increased over a wider time range (44 features (25%) at two time points, 21 features (12%) at three time points, 6 features (3.5%) at four time points; Figure 6B, Supplementary Table 3).

### Amount of Specific Compounds of the Plipastatin Family Is Increased in the Co-cultivation of *S. plymuthica* 4Rx13 With *B. subtilis* B2g

**Figure 7:**
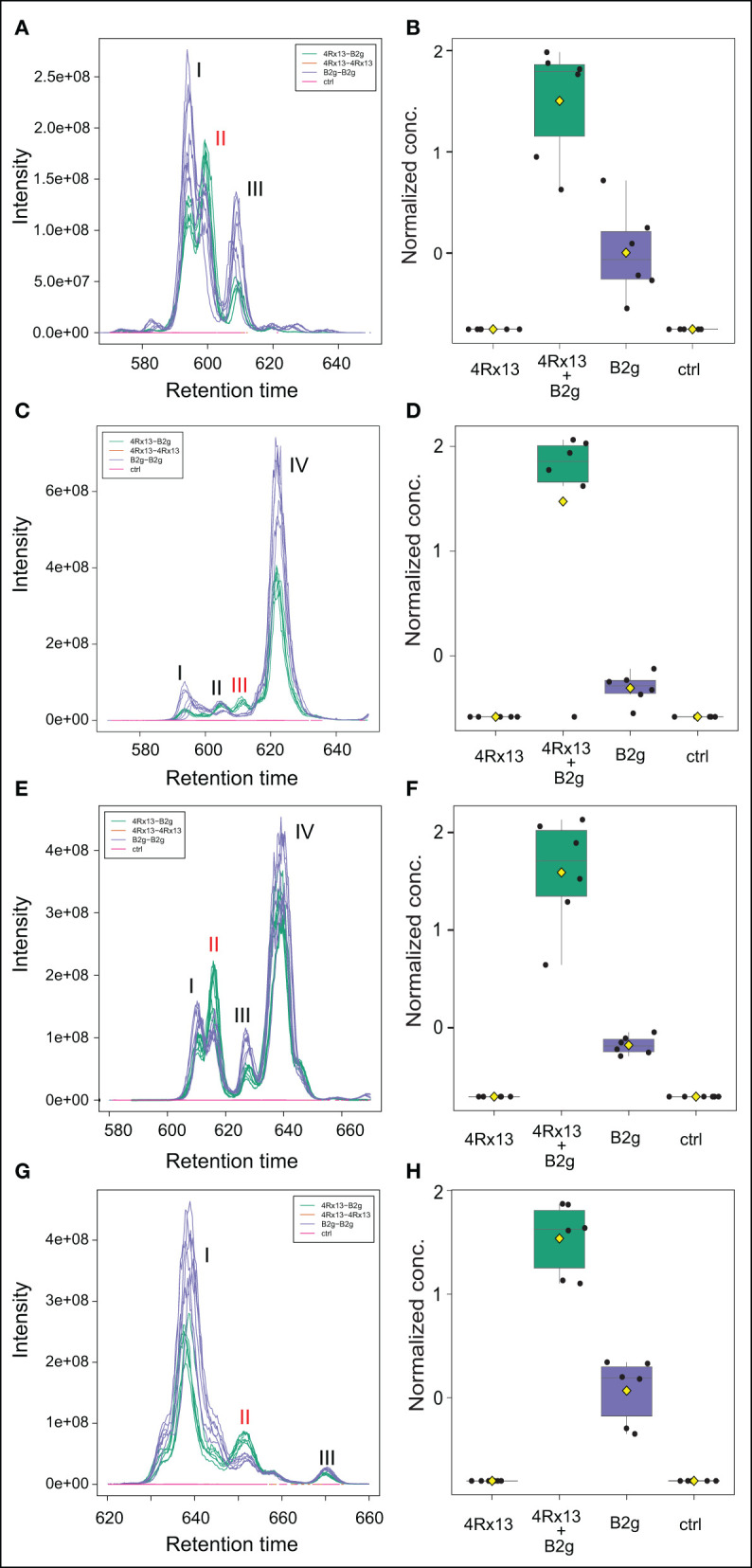
Specific plipastatin isomers are increased in co-cultivation of *S. plymuthica* 4Rx13 with *B. subtilis* B2g. Computed extracted ion chromatograms (respective *m/z* of every plipastatin was extracted and plotted using XCMS, cEIC) and normalized concentration of plipastatins at day 21 of co-cultivation of *S. plymuthica* 4Rx13 with *B. subtilis* B2g (4Rx13+B2g), mono-cultivation of respective species (4Rx13; B2g) and medium control (ctrl). **(A)** cEIC plipastatin [M+2H]^2+^ *m/z* 725.3984. **(B)** Normalized conc. of isomer II (A1). **(C)** cEIC plipastatin [M+2H]^2+^ *m/z* 732.4052. **(D)** Normalized conc. isomer III (A1 and B2). **(E)** cEIC plipastatin [M+2H]^2+^ *m/z* 739.4138. **(F)** Normalized conc. of isomer II (B1). **(G)** cEIC plipastatin [M+2H]^2+^ *m/z* 746.4209. **(H)** Normalized conc. of isomer II (B2).

Next, we were interested in compounds corresponding to the mass features that are more pronounced in the co-cultivation of *S. plymuthica* 4Rx13 and *B. subtilis* B2g. We focused on mass features, which showed most prominent correlation patterns over a wider cultivation range (Figure 7). Although we used the respective algorithm to demask isotopologues and adducts, admittedly not every single mass feature represents one individual compound. Especially, isotopologues of doubly protonated compounds were still observed, however, since these features correlated with the monoisotopic mass features, their presence supported the following results. Accurate masses of certain increased mass features corresponded to doubly protonated ions belonging to four compounds of the plipastatin family ([M+2H]^2+^ *m/z* 725.39841, [M+2H]^2+^ *m/z* 732.40524, [M+2H]^2+^ *m/z* 739.41381, [M+2H]^2+^ *m/z* 746.42243; Table 1). Verification of the statistical calculation was performed by extraction and plotting the respective *m/z* of every feature using XCMS package in RStudio (Figure 7). Interestingly, only specific homologs of the plipastatins were increased in co-cultivation. By determination of accurate masses of specific reporter ions in the acquired tandem mass spectra (Pathak et al., 2012, Kaki et al., 2020), we further characterized the isomers according to presence of specific amino acids in their peptide sequence and classified them into class A and B (Supplementary Figure 2, Table 1, Supplementary Table 4). Of those isomers, plipastatin A1 ([M+H]^+^ *m/z* = 1448.7797), plipastatin B1 ([M+H]^+^ *m/z* = 1476.811) and plipastatin B2 ([M+H]^+^ *m/z* = 1490.8266) were increasingly produced by *B. subtilis* B2g due to co-cultivation with *S. plymuthica* 4Rx13. The peak corresponding to the co-cultivation triggered plipastatin with [M+H]^+^ *m/z* = 1462.7954 showed class A and B reporter ions in its mass spectrum indicating two co-eluting plipastatin isomers. Therefore, we currently assume an increased production of either one or both of these isomers (Supplementary Figure 2). Future investigations including synthesis are needed to unambiguously verify these results, as well as to determine the exact fatty acid sequence for all four increased plipastatins.

**Figure 8:**
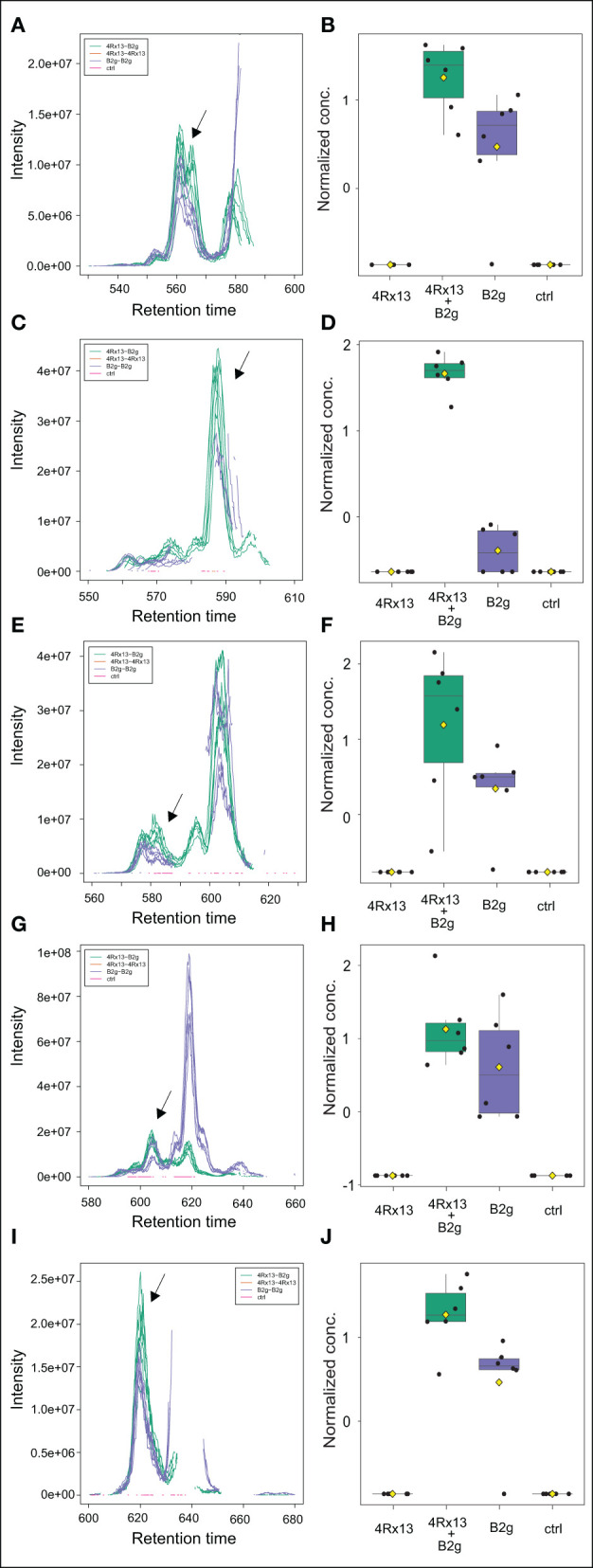
Mass features corresponding to specific isomer of putative plipastatin variants are more pronounced in co-cultivation of *S. plymuthica* 4Rx13 with *B. subtilis* B2g. Computed extracted ion chromatograms (respective *m/z* of every plipastatin variant was extracted and plotted using XCMS, cEIC) and normalized concentration of plipastatins at day 21 of co-cultivation of *S. plymuthica* 4Rx13 with *B. subtilis* B2g (4Rx13+B2g), mono-cultivation of respective species (4Rx13; B2g) and medium control (ctrl). **(A)** cEIC plipastatin variant 1 [M+2H]^2+^ *m/z* 734.4037. **(B)** Normalized conc. of arrow labeled isomer. **(C)** cEIC plipastatin variant 2 [M+2H]^2+^ *m/z* 741.411. **(D)** Normalized conc. of arrow labeled isomer. **(E)** cEIC plipastatin variant 3 [M+2H]^2+^ *m/z* 748.4184. **(F)** Normalized conc. of arrow labeled isomer. **(G)** cEIC plipastatin variant 4 [M+2H]^2+^ *m/z* 755.4268. **(H)** Normalized conc. of arrow labeled isomer. **(I)** cEIC plipastatin variant 5 [M+2H]^2+^ *m/z* 762.4346. **(J)** Normalized conc. of arrow labeled isomer.

**Figure 9:**
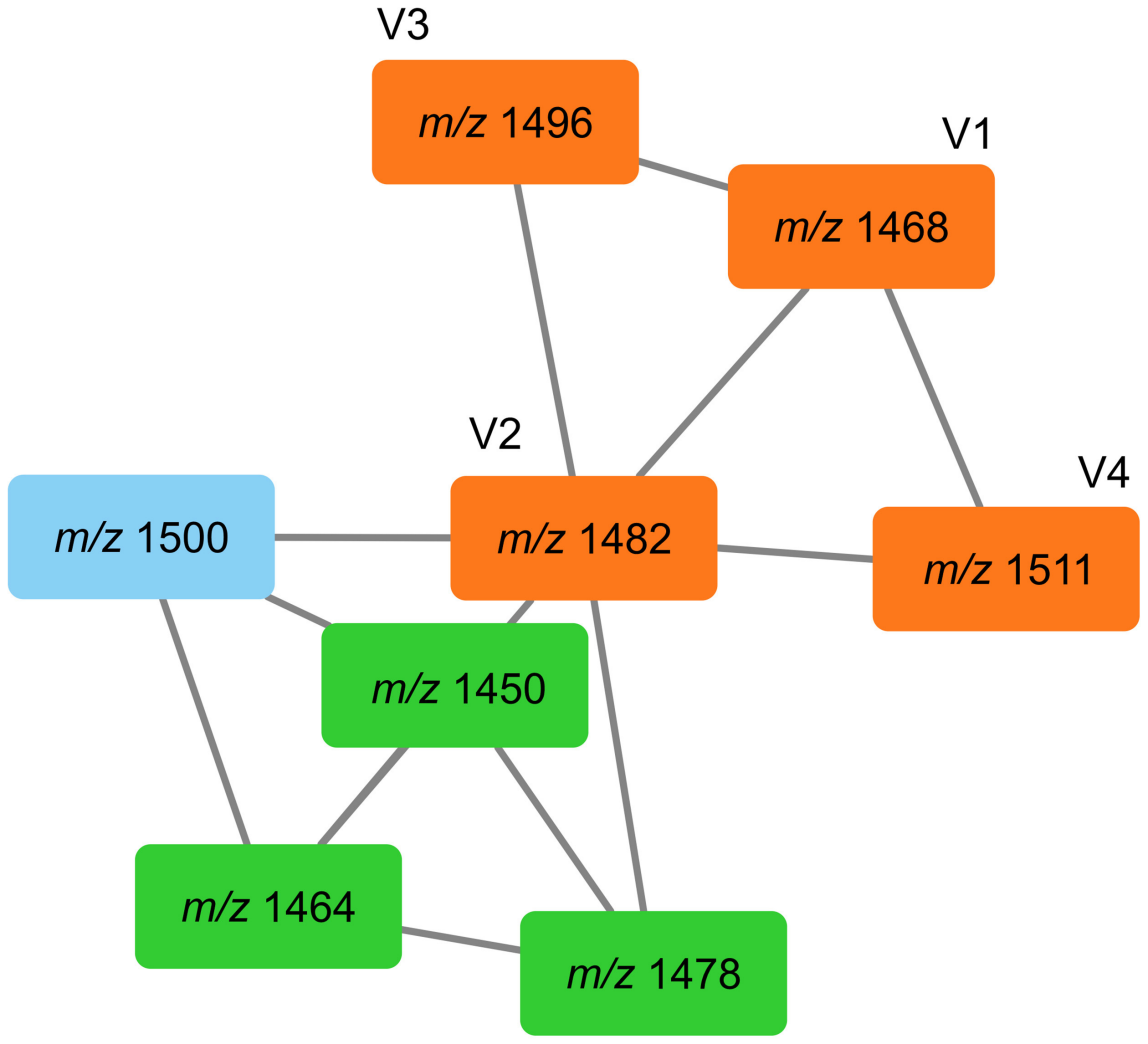
Tandem mass spectra similarities of plipastatins and plipastatin variants V1–V4 shown GNPS. Searched features (V1–V4) are represented in orange boxes, known plipastatins are represented in green boxes, *m/z* in boxes correspond to nominal masses of precursor ions.

In addition, we detected further co-cultivation increased doubly protonated features at [M+2H]^2+^ *m/z* 734.40378 (C_71_H_110_N_12_O_21_, Δppm 1.5), [M+2H]^2+^ *m/z* 741.41101 (C_72_H_112_N_12_O_21_, Δppm 0.7), [M+2H]^2+^ *m/z* 748.41870 (C_73_H_114_N_12_O_21_, Δppm 0.5), [M+2H]^2+^ *m/z* 755.42680 (C_74_H_116_N_12_O_21_, Δppm 0.8), [M+H]^2+^ *m/z* 762.43459 (C_75_H_118_N_12_O_21_, Δppm 0.7) (Table 1). The accurate masses of these features showed a consecutive mass difference of *m/z* 18.01054 corresponding to H_2_O (Δppm 1.4) to the known plipastatins indicating that these co-cultivation increased features correspond to plipastatin variants (labeled as V1-V5). For the proposed chemical formulas, we did not find literature references and no entries in PubChem (https://pubchem.ncbi.nlm.nih.gov/) suggesting putative novel plipastatin variants. Again, we verified the data by extraction and plotting of the respective *m/z* (Figure 8). As shown in Figure 8, the monoisotopic masses of each feature presented at least three to four isomers. Again, only certain isomers were increasingly produced in response to the interaction. Due to the low abundance of the putative plipastatin variants, the MS/MS spectra are so far insufficient for precise structure elucidation. However, molecular networking using these poor quality mass spectra already indicated a clear connection for four of the searched features to members of the known plipastatins (Figure 9).

### Intraspecific Co-cultivation of *S. plymuthica* 4Rx13 and *S. plymuthica* AS9 Revealed a Distinct Interaction Zone and Showed Distinct Metabolic Profiles Compared to Mono-cultures

Next, we were interested in the intraspecific interaction potential of *S. plymuthica* 4Rx13 and performed similar interaction assays with the rhizobacterial *S. plymuthica* isolate AS9.

**Figure 10:**
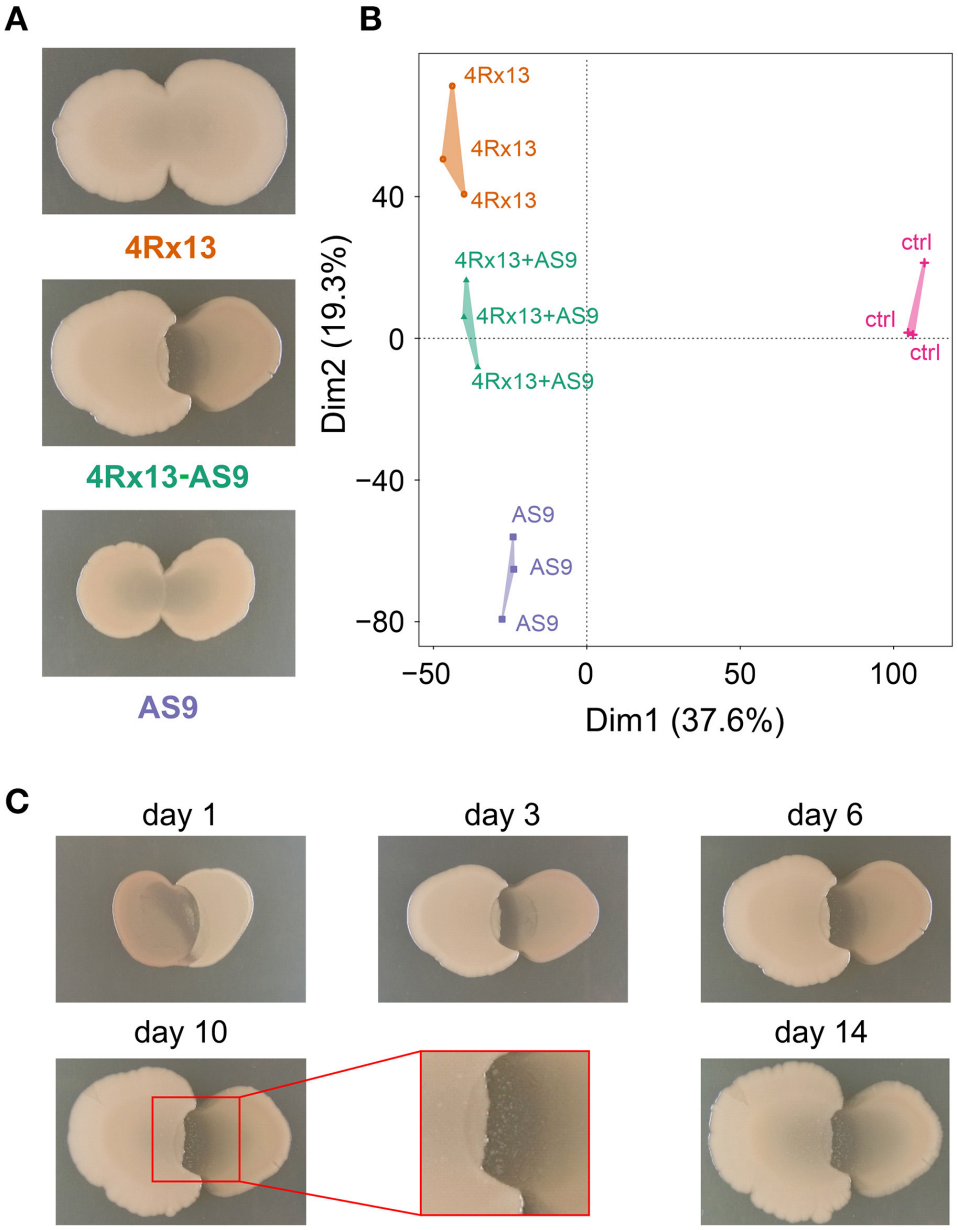
Intraspecific interaction of *S. plymuthica* 4Rx13 and AS9 on day 6 of cultivation. **(A)** representative photographs of self-paired and co-culture **(B)** PAM-Clusterplot (computation of 4 clusters); 4Rx13 correspond to mono-cultivation of *S. plymuthica* 4Rx13 (red), 4Rx13 vs. AS9 (green), AS9 monoculture (blue), ctrl (control, extracted non-inoculated agar, violet). **(C)** Co-cultivation during growth including a zoom out of interaction zone (red box).

Co-cultivation led to a clear and distinct interaction zone, which was not observed in self-paired cultures (Figure 10A). Images of the interaction zone indicate that both partners are suppressed by the interaction. Development of this zone starts at day 1 of interaction (Figure 10C). At day 6, small colony-like spots appeared in the interaction zone.

After metabolite extraction, XCMS analysis and data processing, the PAM clustering plots from cultures at day 6 of cultivation showed four distinct clusters [Figure 10B, Cluster 1–4Rx13 mono, 2–4Rx13+AS9 interaction, 3—AS9 mono, 4—ctrl (agar)]. Similar to the 4Rx13 – B2g interaction, this clear separation was not observed at day 1 and 3 of cultivation (Supplementary Figure 3). The clear separation was only present at day 6 and 7 of cultivation. At day 10, the PAM algorithm calculated one bigger cluster including mono-cultivated *S. plymuthica* 4Rx13 and co-cultivated *S. plymuthica* 4Rx13/AS9 metabolites, which was also observed in the further stages of cultivation. At day 28 the metabolic differences are completely abolished. These results suggest that the metabolic profiles of the co-cultures differ at specific culture stages from the metabolic profiles of the mono-cultures. During later growth, the metabolites released by *S. plymuthica* 4Rx13 seem to dominate the metabolic profiles of the co-culture.

### Intraspecific Interaction of *S. plymuthica* 4Rx13 and *S. plymuthica* AS9 Showed Altered Mass Features Compared to Mono-cultivated Species

**Figure 11:**
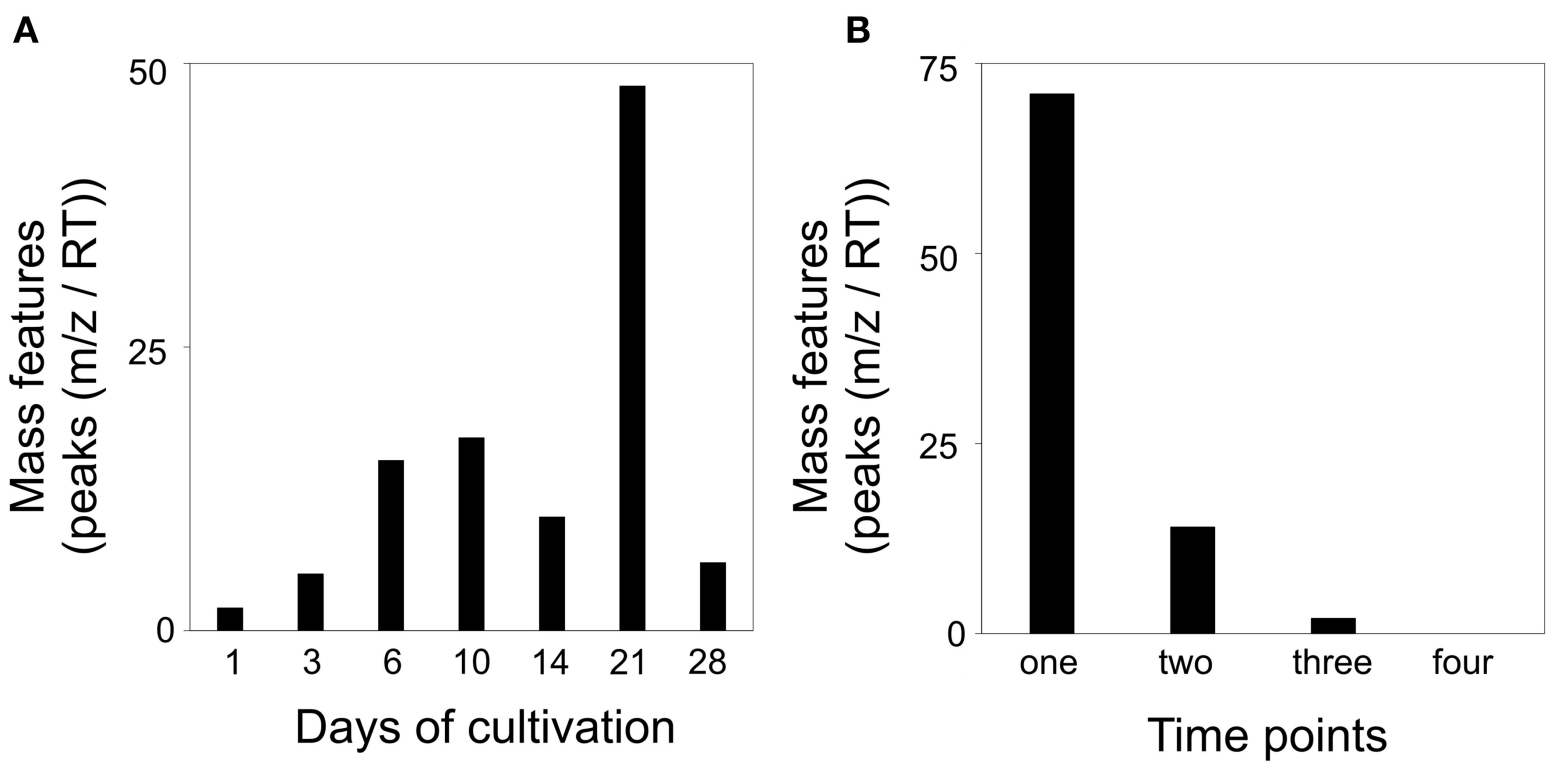
Altered mass features [peaks (*m/z*/RT)] in co-cultivation of *S. plymuthica* 4Rx13 and *S. plymuthica* AS9 in comparison to mono-cultivated strains and medium control (correlation threshold > 0.8). **(A)** Number of co-cultivation correlating mass features in relation to each day of cultivation (extracts were sampled at day 1, 3, 6, 10, 14, 21, and 28 of mono and co-cultivation of *S. plymuthica* 4Rx13 and AS9). **(B)** Frequency of occurrence of each co-cultivation correlating mass feature.

Next, we analyzed for patterns that were differentially featured in intraspecific co-cultivation of interacting *S. plymuthica* 4Rx13 with *S. plymuthica* AS9 in comparison to both mono-cultivated strains and control. We raised the threshold of the correlating patterns to 0.8 due to the lower number of replicates (*n* = 3). In total, we found in interaction 87 differentially-induced mass features (Supplementary Tables 5, 6). After a constant increase from day 1 until day 10, followed by a drop at day 14, the highest change was observed at day 21 of interaction (Figure 11A). At day 28 of interaction, the altered mass features decreased again. No single mass feature was increased over the whole time range of co-cultivation. Seventy-one features (81.6 %) differed at one single time point only, while 16 features (18.4%) were increased over a wider time range (14 features (16.1%) at two time points, 2 features (2.3%) at three time points; Figure 11B, Supplementary Table 6).

### Several Short Peptides Are Increased in Co-cultivation of *S. plymuthica* 4Rx13 With *S. plymuthica* AS9

**Figure 12:**
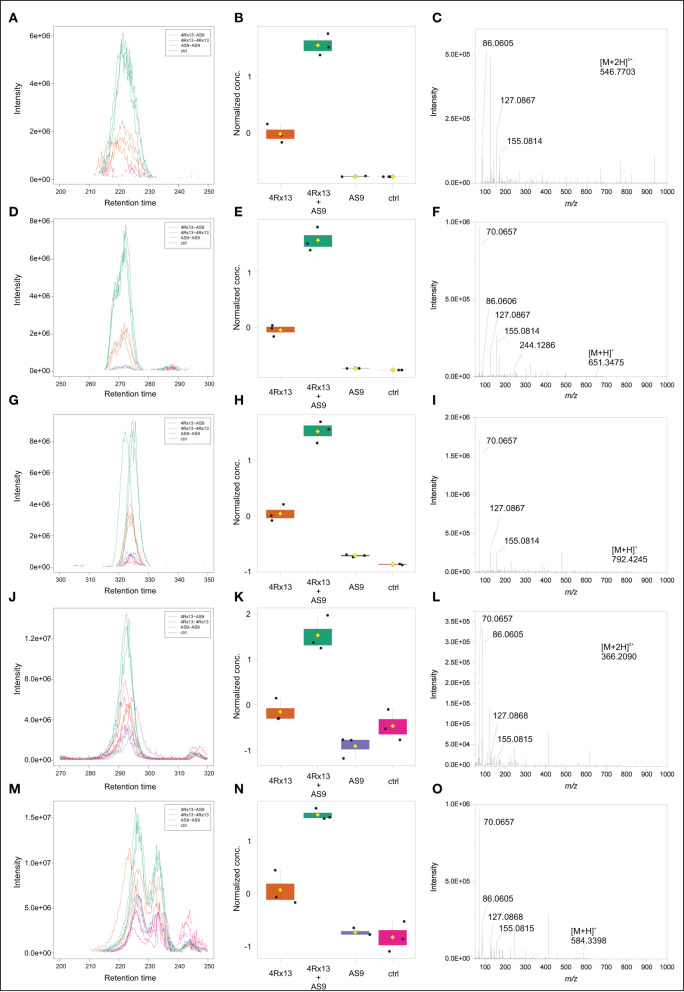
Intraspecific co-cultivation triggered putative peptides. cEIC **(A)**, Normalized conc. **(B)**, MS/MS **(C)** of [M+2H]^2+^ *m/z* 546.7703. cEIC **(D)**, Normalized conc. **(E)**, MS/MS **(F)** of [M+H]^+^ *m/z* 651.3475. cEIC **(G)**, Normalized conc. **(H)**, MS/MS **(I)** of [M+H]^+^ *m/z* 792.2445. cEIC **(J)**, Normalized conc. **(K)**, MS/MS **(L)** of [M+H]^+^ *m/z* 366.2090. cEIC **(M)**, Normalized conc. **(N)**, MS/MS **(O)** of [M+H]^+^ *m/z* 584.3398.

**Figure 13:**
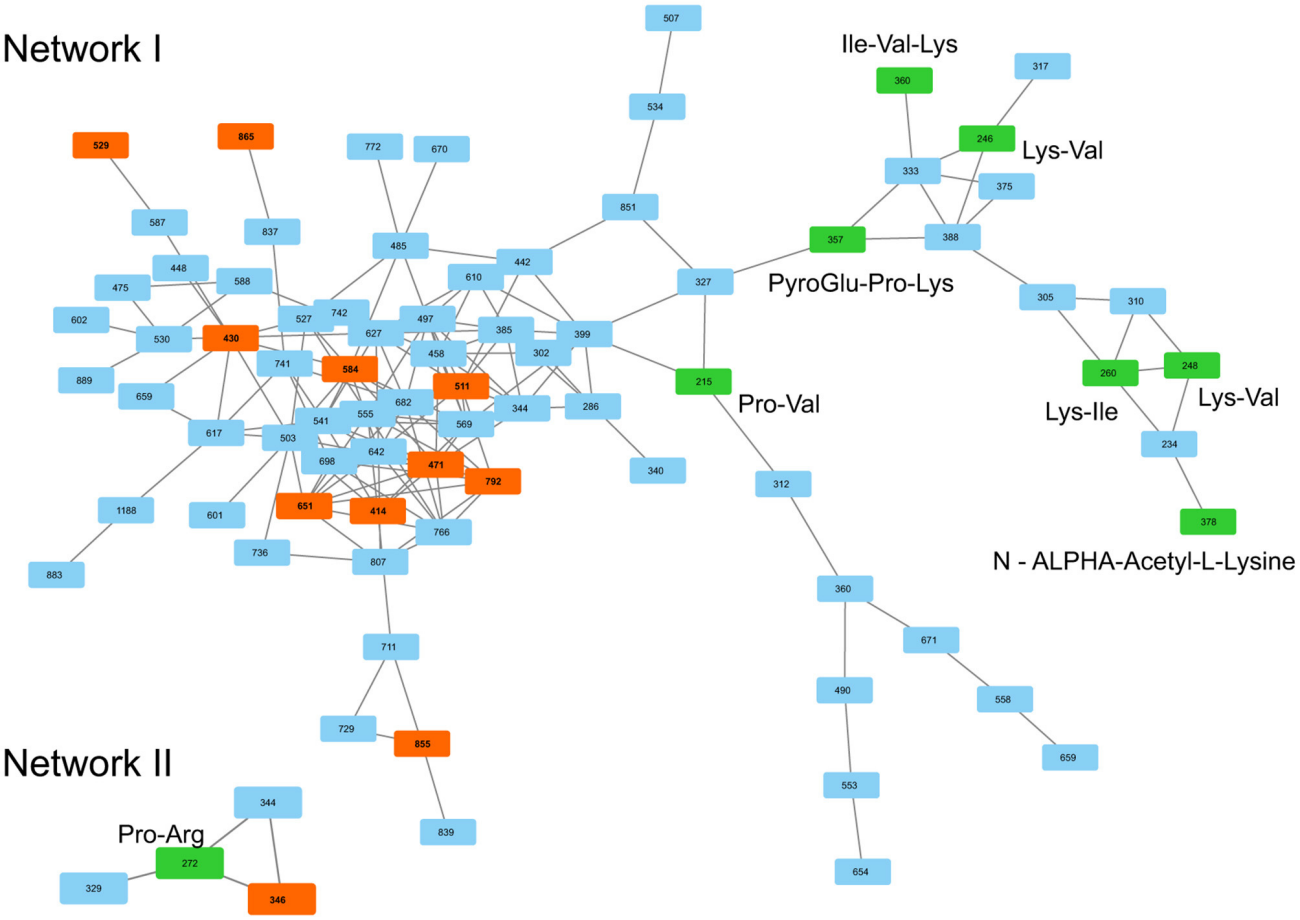
MS/MS similarities of *S. plymuthica* 4Rx13 and AS9 intraspecific co-cultivation triggered mass features shown by GNPS. Searched features are represented in red boxes, previously annotated peptides are represented in green boxes. *m/z* in boxes correspond to nominal masses.

Across all co-cultivation correlating mass features, we focused on a group of single and doubly protonated features that showed similar tandem mass spectra, exemplarily shown for five prominent mass features in Figure 12. These features were mostly produced by *S. plymuthica* 4Rx13 mono-cultures, too, however in lower amounts (Figure 12, Supplementary Figure 4). Features that have been found to be increased in co-cultivation and which are also produced by both mono-cultivated strains were excluded in this studies (see Supplementary Figures 4D,E,G,Q,T,W). For some features, two isomers were detected (Figure 12M, Supplementary Figures 4B,D–F,H–L,O). Mass range of all other features along with occurring fragments at *m/z* 70.0657, 86.0605, 127.0867, and 155.0814 indicate related short proline-containing peptides. The high spectral similarities of tandem mass spectra and the peptide prediction was strengthened for several of these features by a molecular networking approach (Figure 13). Features with [M+H]^+^ *m/z* 414.2352, 430.2302, 471.2568, 511.2883, 529.2985, 584.3408, 651.3471, 792.4258, 865.4427 clustered in a network containing the previously annotated small peptides Lys-Ile, Ile-Val, Ile-Val-Lys, Lys-Val, PyroGlu-Pro-Lys and Pro-Val, while [M+H]^+^ *m/z* 346.2085 was networking with a Pro-Arg peptide. Fragmentation pattern analysis using Sirius was further used to putatively determine chemical formulae from single protonated ions only, since doubly protonated features are not supported by Sirius (Table 2). Thereby, nine out of eleven selected chemical formulae were computed at first rank with seven features showing a Sirius scores above 80 %. For two features, we selected the second ranked formula due to unlikely elemental composition of the first rank formula. PubChem search for these chemical formulae revealed several possibilities of short peptides with up to 8 amino acids. Future structural investigations are needed to confirm these assumptions, as well as to elucidate the relationship between these individual peptides.

**Table 2:** Putative chemical formulae of peptides increased in co-cultivation of *S. plymuthica* 4Rx13 and AS9 (* rank 2 in Sirius calculation).

***m/z* detected [M+H]^**+**^**	**RT (sec)**	**Chemical formula**	**Sirius score (%)**	**Δppm**
346.2085	250	C_14_H_27_N_5_O_5_	99.982	0.05
404.2143	238	C_16_H_29_N_5_O_7_	96.948	0.76
471.2568	243	C_20_H_34_N_6_O_7_	91.21	1.41
529.2985	303	C_23_H_40_N_6_O_8_	85.324	0.91
558.2884	253	C_23_H_39_N_7_O_9_	80.875	0.61
565.263	277	C_25_H_36_N_6_O_9_	87.165	2.4
584.3408	233	C_26_H_45_N_7_O_8_	82.769	0.95
651.3471	272	C_29_H_46_N_8_${\text{O}}_{9}^{*}$	34.999	1.6
792.4258	324	C_36_H_57_N_9_O_11_	49.544	1.0
865.4427	294	C_38_H_60_N_10_${\text{O}}_{13}^{*}$	25.154	1.5
1033.533	303	C_51_H_72_N_10_O_13_	18.477	2.1

## Discussion

Bacteria are not solitary organisms but rather, maintain complex interactions within microbial communities to ensure their survival (Nadell et al., 2016; Yanni et al., 2019). Bacterial metabolism differ in these communities due to competitive and cooperative actions compared to single existence (Little et al., 2008). As a result, some metabolites that are not produced by solitary living bacteria, or are produced only weakly, are assumed to be biosynthesized more abundantly by the same bacteria within communities, e.g., toxins, antibiotics or antifungal compounds. In contrast, when living in a community there is no need to synthesize metabolites equally to solitary life, since metabolites can be exchanged between members of the community (Phelan et al., 2012). To understand the basic principles of the relations between the individuals of bacterial species within these communities, we need to apply a wide range of experimental holistic and reductionistic approaches. Here, we evaluated metabolic profiling using multivariate analyses combined with classical mass spectrometry identification as well as emerging computational identification tools as approaches to study changes in metabolite excretion of the rhizobacteria *S. plymuthica* 4Rx13, *B. subtilis* B2g and *S. plymuthica* AS9 due to bacterial interaction.

### Change of Metabolic Status During Cultivation

Our first results demonstrated that changes in metabolism during cultivation of all three tested bacteria, the *S. plymuthica* isolates 4Rx13 and AS9 as well as *B. subtilis* B2g, can be clearly distinguished using metabolic profiling of metabolites released into the agar. These changes are most likely due to the depletion of nutrients during the *in vitro* cultivation on agar (Meyer et al., 2014), but might also be related to biosynthesis of primary and specialized metabolites coordinated to different growth stages (Stein, 2005; Sanchez and Demain, 2008; Raaijmakers et al., 2010). The observed decrease of variations late in cultivation time (day 28 and day 30) might indicate a reached steady state of released metabolites, however, cultivation over a longer period of time is needed to clarify this hypothesis. Future investigations will tackle the exact background of the observed alterations. However, taking these results into consideration, we might already need to reconsider the currently applied co-cultivation assays, which are, for instance, performed by placing droplets of two liquid cultures at the same time point on solid media or in more recent approaches using microfluidic devices (Liu et al., 2010; Park et al., 2011; Watrous et al., 2012; Burmeister et al., 2019). Although these assays are very useful for evaluating molecular differences in interacting microbial communities (Watrous et al., 2012), we might miss a lot of interplay due to non-optimized time windows of co-cultivation. Inoculations of two bacterial strains each at a different time point might cause various effects always depending on the current metabolic status of each strain. More dynamic studies in which the bacteria are grown separately from each other over a certain time before allowing them to interact could verify this issue.

### Inter- and Intraspecific Variations of Released Metabolites

Using metabolite profiling, we found an interspecific variation between metabolites of *S. plymuthica* 4Rx13 and *B. subtilis* B2g. This considerable interspecific contrast in which both strains showed almost similar numbers of different features was expectable, as the metabolism and physiology can be assumed to vary greatly due to the phylogenetic and morphological difference between *S. plymuthica* and *B. subtilis*. Chubukov and Sauer, for instance, found significant differences between metabolic phenotypes of model bacteria *E. coli*, which like *S. plymuthica* belong the family of *Enterobacteriaceae*, and *B. subtilis* when characterizing their stationary-phase metabolites (Chubukov and Sauer, 2014). Furthermore, *B. subtilis* is in general known for its production of different secondary metabolites including the lipopeptides surfactin, iturin, and plipastatin (Kakinuma et al., 1969; Peypoux et al., 1981; Nishikiori et al., 1986; Kluge et al., 1988; Stein, 2005), which are not described for *Serratia* species. Therefore, it was no surprise that exclusive lipopeptide production (surfactin, bacillomycin D and plipastatin) by *B. subtilis* B2g were partly responsible for the interspecific differences, similarly to Oocydin A and Serratiochelin that were exclusively produced by *S. plymuthica* 4Rx13. We have to admit that the extracts of both strains contained more promising secondary metabolites, which are currently under investigation.

More remarkable was the intraspecific variation we observed between excreted metabolites of the *S. plymuthica* isolates 4Rx13 and AS9. These high variations were unexpected since the main primary metabolism can be assumed to be similar, when related isolates from the same species that are isolated from similar rhizosphere habitats grow under identical *in vitro* conditions. With twice the number of exclusively—or increasingly produced features *S. plymuthica* 4Rx13 showed even higher intraspecific variation to *S. plymuthica* AS9 compared to interspecific variation to *B. subtilis* B2g. Of the differing features secondary metabolites produced by both isolates reflected the intraspecific metabolic differences. While *S. plymuthica* 4Rx13 solely releases the haterumalide Oocydin A, *S. plymuthica* AS9 exclusively produces Prodigiosin, a putative hexadecylbutanediamine structure and several members of the Serratomolide family. The siderophore Serratiochelin was released by both strains, however, in higher amounts by *S. plymuthica* 4Rx13. The three secondary metabolites Serratiochelin A, Oocydin A and Prodigiosin have been previously described in *S. plymuthica* isolates (Grimont and Grimont, 2004; Matilla et al., 2012; Cleto et al., 2014). The production of the antimicrobial biosurfactant lipopeptide Serratomolide isomers has so far been found in the genus *Serratia*, however, only in two species *S. marcescens* and *S. surfactantfaciens* and not yet in *S. plymuthica* (Wassermann et al., 1961; Clements et al., 2019). Recent genome comparison via antiSMASH software revealed that *S. plymuthica* AS9 carries the Serratomolide *swrW* gene (Marques-Pereira et al., 2020). Our findings now demonstrate the ability of *S. plymuthica* AS9 to produce Serratomolide biosurfactants, although further profound structural characterization of individual isomers is needed to give an insight into the full lipopeptide profile. Since the found hexadecylbutanediamine structure shows similarities to the Zeamines, which are polyamine-polyketide-non-ribosomal peptide antibiotics that are strongly active against various Gram-positive and Gram-negative bacteria by affecting the integrity of cell membranes; most probably due to interaction of the positively charged amino groups with the polyanionic LPS (Masschelein et al., 2015), their identity also needs structural confirmation in future. Furthermore, the remaining features that are exclusively released by *S. plymuthica* 4Rx13 and AS9 should be explored in more detail. These analyses in combination with continuing metabolite profiling of more *S. plymuthica* isolates will answer the question whether these high intraspecific variations represent a general phenomenon within the species *Serratia plymuthica*.

### Interspecific Interaction Between *S. plymuthica* 4Rx13 and *B. subtilis* B2g

Preliminary phenotypic data of the interaction between the two rhizobacteria *S. plymuthica* 4Rx13 and *B. subtilis* B2g indicated an immense potential of inter-organismic crosstalk (Kai and Piechulla, 2018). Present PAM-clustering data show that the profiles of metabolites released during this interaction consistently vary from the profiles of both mono-cultivated bacteria. At the beginning of cultivation PAM-cluster analysis did not reveal clear separation. Since we found *B. subtilis* B2g mono-cultures clustering with non-inoculated agar extracts at day 1 and 3 of cultivation, we assume that is due to low concentration of released metabolites by *B. subtilis* B2g caused by their slower growth compared to *S. plymuthica* 4Rx13 (Kai and Piechulla, 2018). Similar results were observed by Zhou et al. (2011), who monitored the interaction of *Bacillus megaterium* and *Ketogulonicigenium vulgare* over 72 h using PCA. They found intracellular metabolites of co-cultures at a shorter distance to the mono-cultures at 48 h than at 72 h (Zhou et al., 2011). The fluctuation of PAM-clustering that we observed late in growth (day 28 and 30) indicates an decreasing variation of metabolite content between mono- and interacting cultures with increasing cultivation time. More precisely, the data suggest that metabolites, which are produced by *S. plymuthica* 4Rx13 might shape the metabolite content of an established structured co-culture between *S. plymuthica* 4Rx13 and *B. subtilis* B2g. Future investigations in which both strains interact with each other over a longer period of time can clarify this hypothesis.

In recent literature it is described that interspecies crosstalk can trigger “cryptic” genes to produce novel natural products (Scherlach and Hertweck, 2009; Ochi, 2017; Van Bergeijk et al., 2020). So far, we did not detect features that were solely present in co-cultivation indicating that the distinct multivariate cluster separation based most likely on altered production and consumption of metabolites by the co-cultured strains in comparison to the mono-cultivated ones rather than the release of “cryptic” encoded natural products. The number of metabolites varying between mono- and co-cultivation increased with the progressing of cultivation, indicating an enhanced interaction potential with establishment of the co-cultivation. Within this study we focused on exploring the most prominent metabolite differences occurring due to interaction. One class of metabolic features, which differed in later stages of interaction between *S. plymuthica* 4Rx13 and *B. subtilis* B2g belonged to compounds of the plipastatin family. Plipastatins are bioactive lipopeptides produced by isolates of *Bacillus subtilis* isolates (Nishikiori et al., 1986; Ongena et al., 2007; Hussein, 2019), mainly known for their striking antifungal activity (Kaspar et al., 2019; Kiesewalter et al., 2021). They occur as various isomers characterized by different structures (A1, A2, B1, and B2; Kaspar et al., 2019) of whom only specific isomers were increasingly produced by *B. subtilis* B2g in the co-cultivation with *S. plymuthica* 4Rx13. Interestingly, further increased features were putatively identified using accurate mass measurements and mass spectral molecular networking as specific isomers of not yet described plipastatin variants. These variants we would probably have overlooked, if they had not been triggered by co-cultivation. The response in production of only specific isomers both of plipastatins as well as plipastatin variants to the interplay with *S. plymuthica* 4Rx13 indicates either that specific precursors amino—or fatty acids are more abundant or a specific regulation by *B. subtilis* B2g due to the co-cultivation. Full characterization of the isomers in order to evaluate the exact structure is needed to initiate answering these questions. So far, an interaction-increased production of lipopeptides upon perception of bacterial competitors has not been shown (Andric et al., 2020). Recent data, however, has shown that fungal interaction triggered *B. subtilis* NCIB3610 to activate the regulator SigB leading to an enhanced surfactin production (Bartolini et al., 2019). Interestingly, the observed induced production does not generally apply for the lipopeptides produced by *B. subtilis* B2g since compounds of the surfactin and bacillomycin D family did not show an increase during interaction. That plipastatins are increasingly produced is thereby especially interesting, since plipastatins are so far described as antifungal metabolites, however, Raaijmakers and colleagues already stated that “lipopeptides might have different natural roles, some of which may be unique to the producer” (Raaijmakers et al., 2010). The natural role of *B. subtilis* B2g plipastatin overproduction due to interplay with *S. plymuthica* 4Rx13 needs to be investigated in future.

### Intraspecific Interaction Between *S. plymuthica* 4Rx13 and *S. plymuthica* AS9

The intraspecific co-cultivation of *S. plymuthica* 4Rx13 and AS9 was also characterized by a distinct interaction zone indicating high potential of intraspecific antagonism between both partners. Thereby, the intraspecific interplay between both isolates led to distinct metabolic profiles with a high number of differentially-featured masses compared to mono-cultivated species. Interestingly, several very short putatively proline-containing peptides were found to be more pronounced in co-cultivation compared to mono-cultivated strains. Short proline-rich antimicrobial peptides (PrAMPs), mostly isolated from insects, are promising antibiotics showing a broad range of antibacterial activities against several Gram-positive and Gram-negative bacteria (Otvos, 2002; Cardoso et al., 2019). However, the peptides observed in this study are shorter with presumably 2–8 amino acids compared to the PrAMPs mostly occurring with more than 18 amino acid residues (Otvos, 2002). In order to evaluate whether the observed peptides have antibacterial activity we firstly need their complete structural characterization. This characterization will also help to evaluate the identity of the producing partner, since, although our results indicate that the main source of these peptides seems to be *S. plymuthica* 4Rx13, we cannot exclude an induction of peptide production in *S. plymuthica* AS9 due to interplay with *S. plymuthica* 4Rx13. Currently, we also do not know whether these peptides are actively released or passively leak through membranes that have lost their integrity due to antagonism. It is also possible that higher proteins or peptides were increasingly degraded by proteases during interaction leading to shorter peptides since *S. plymuthica* AS9, for instance, showed proteolytic activity in earlier studies (Alström, 2001). Future studies should tackle these questions of increased amounts of peptides and clarify their putative function in intraspecific crosstalk.

## Conclusion

Due to the complexity of rhizobacterial communities investigations of interactive processes within this bacterial network are challenging. Using metabolic profiling, we showed interspecific and intraspecific variations of metabolic profiles and further that the excretion of certain metabolites, i.e., plipastatins and putative proline-containing peptides, can change induced by social interactions between single members of bacterial communities. These results form a basis for biological assays to further investigate the functional role of these interaction-triggered compounds in establishment and maintenance of microbial communities. Furthermore, they can be applied under natural and more realistic conditions, since rhizobacteria also interact with the plant itself and many other members of plant and soil microbiota.

## Data Availability Statement

The datasets presented in this study can be found in online repositories. The names of the repository/repositories and accession number(s) can be found in the article/Supplementary Material.

## Author Contributions

MK conceived, designed the experiments, interpreted the results, and wrote the manuscript. MK, DW, and RM performed the experiments. RM measured the metabolic profiles. MK, RM, and AS analyzed the data. BP discussed preliminary results and the manuscript and provided financial support (BP153/36-1). All authors critically revised and consented to the final version of the manuscript.

## Conflict of Interest

The authors declare that the research was conducted in the absence of any commercial or financial relationships that could be construed as a potential conflict of interest.

## Supplementary Material

Supplementary Figure 1 Distinct metabolic profiles of interspecific co-cultivations of *S. plymuthica* 4Rx13 and *B. subtilis* B2g compared to their mono-cultures. PAM-Cluster plots.

Supplementary Figure 2 Identification of plipastatin isomers using reporter fragment ions corresponding to amino acids at position 6 and 10 in the plipastatin peptide ring. *m/z* 966.45941 and 1080.53918 for Ala^6^/Ile^10^ corresponding to A1, *m/z* 1066.51892 for Ala^6^/Val^10^ corresponding to A2, *m/z* 994.49011 and 1108.56982 for Val^6^/Ile^10^ corresponding to B1, *m/z* 980.47394 and 1094.55005 for Val^6^/Val^10^corresponding to B2.

Supplementary Figure 3 Distinct metabolic profiles of intraspecific co-cultivations of *S. plymuthica* 4Rx13 and *S. plymuthica* B2g compared to their mono-cultures. PAM-Cluster plots.

Supplementary Figure 4 Due to *S. plymuthica* 4Rx13 and AS9 co-cultivation increased features, respective *m/z* of every feature was extracted and plotted (cEIC).

Supplementary Table 1 Lipopeptides produced by *B. subtilis* B2g.

Supplementary Table 2 Differentially induced mass features in *Serratia plymuthica* 4Rx13 interaction with *Bacillus subtilis* B2g compared to mono-cultivated strains and medium control.

Supplementary Table 3 Time points of detection of differentially induced mass features (*m/z*) in *Serratia plymuthica* 4Rx13 interaction with *Bacillus subtilis* B2g compared to mono-cultivated strains and medium control.

Supplementary Table 4 Identification of plipastatin isomers (isomers that are more pronounced in interaction are labeled in red.

Supplementary Table 5 Differentially induced mass features in *Serratia plymuthica* 4Rx13 interaction with *S. plymuthica* AS9 compared to mono-cultivated strains and medium control.

Supplementary Table 6 Time points of detection of differentially induced mass features (*m/z*) in *Serratia plymuthica* 4Rx13 interaction with *S. plymuthica* AS9 compared to mono-cultivated strains and medium control.
