# Supplementary figures and images for: Metabolic Profiling of Rhizobacteria Serratia plymuthica and Bacillus subtilis Revealed Intra- and Interspecific Differences and Elicitation of Plipastatins and Short Peptides Due to Co-cultivation
Source: Front Microbiol. 2021 May 31;12:685224. doi: 10.3389/fmicb.2021.685224 (PMC8200778; doi:10.3389/fmicb.2021.685224)

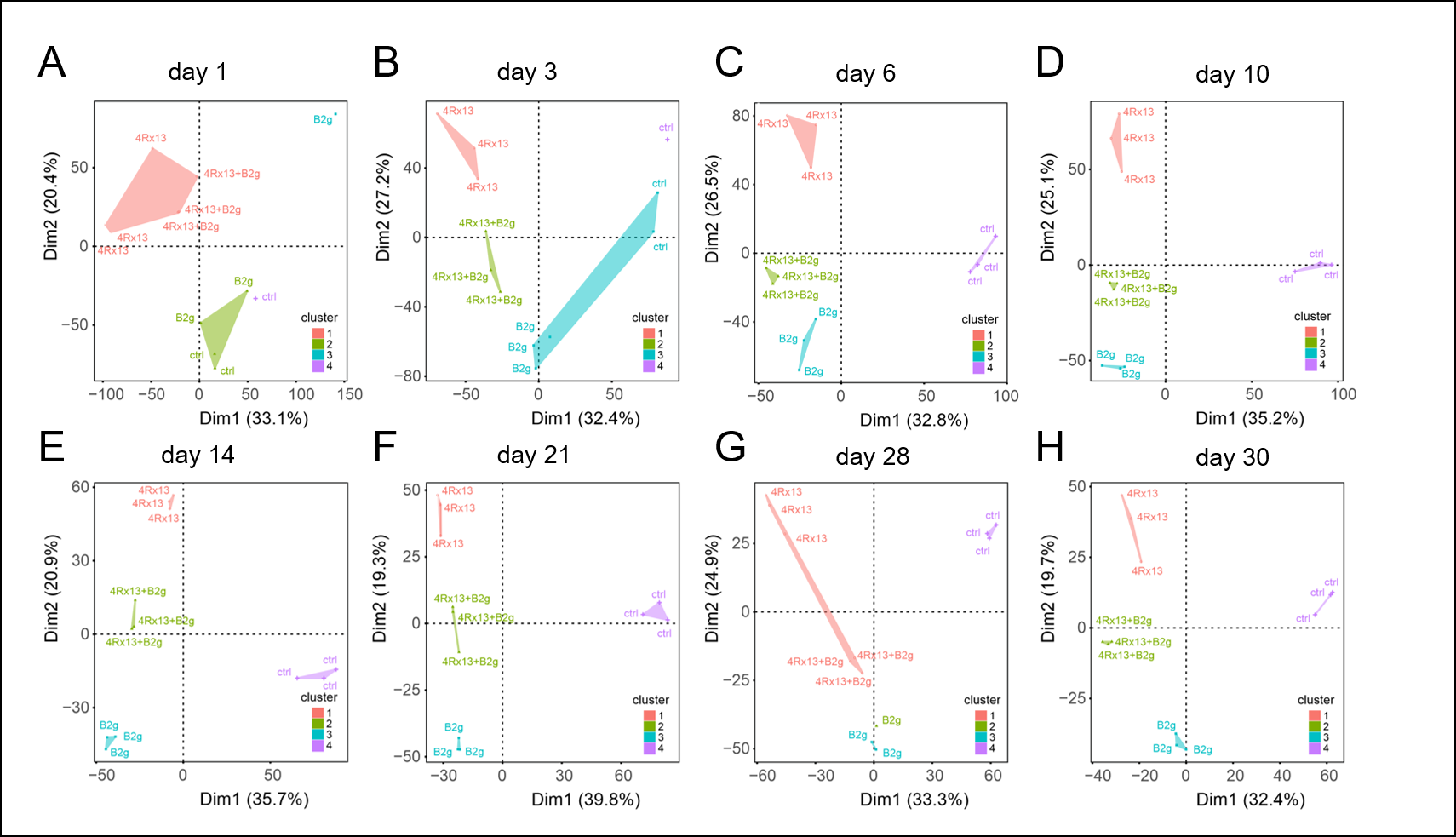

Supplement: Supplementary Figure 1 — Distinct metabolic profiles of interspecific co-cultivations of S. plymuthica 4Rx13 and B. subtilis B2g compared to their mono-cultures. PAM-Cluster plots. [file Image_1.TIF]

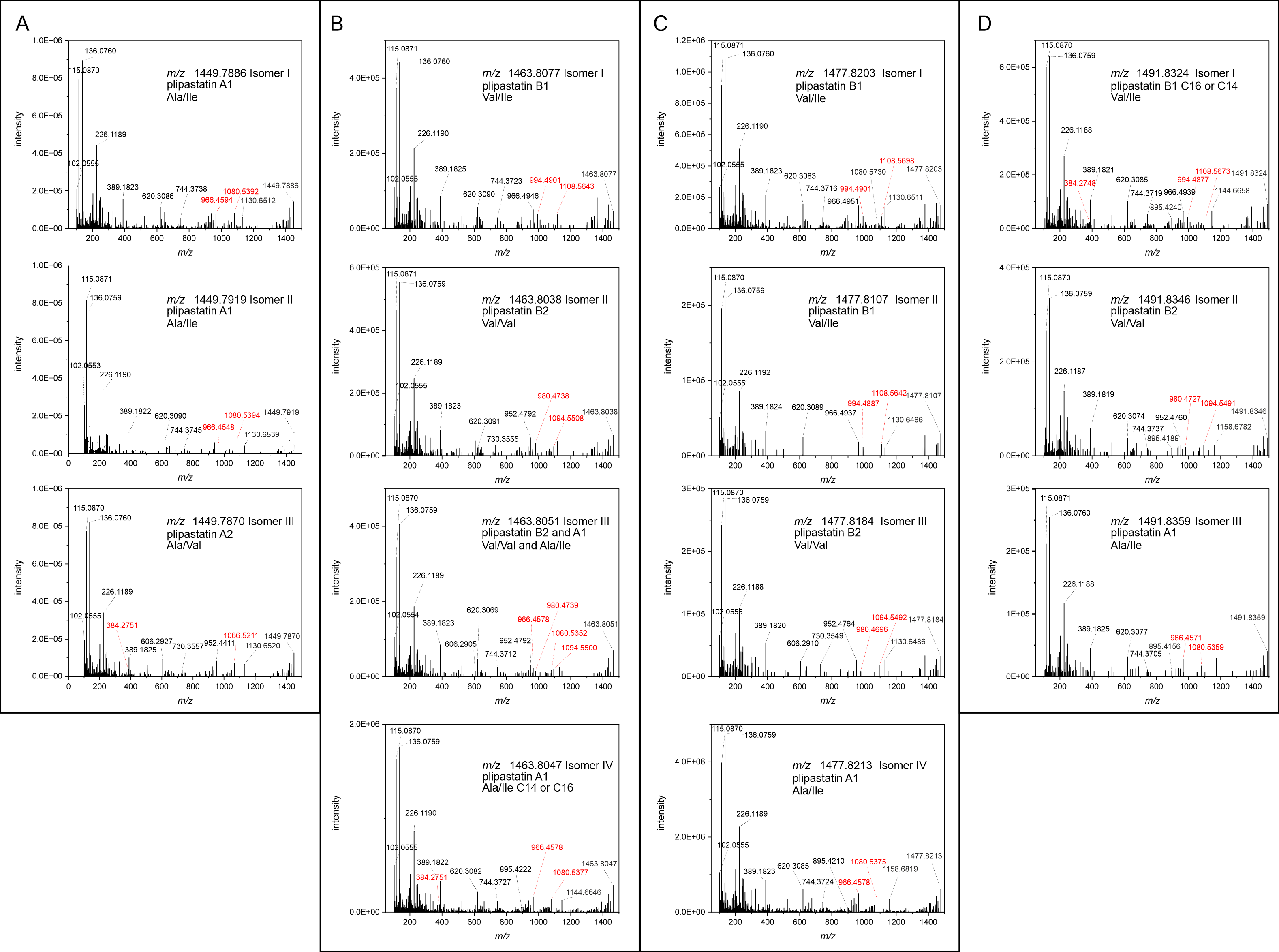

Supplement: Supplementary Figure 2 — Identification of plipastatin isomers using reporter fragment ions corresponding to amino acids at position 6 and 10 in the plipastatin peptide ring. m/z 966.45941 and 1080.53918 for Ala6/Ile10 corresponding to A1, m/z 1066.51892 for Ala6/Val10 corresponding to A2, m/z 994.49011 and 1108.56982 for Val6/Ile10 corresponding to B1, m/z 980.47394 and 1094.55005 for Val6/Val10corresponding to B2. [file Image_2.TIF]

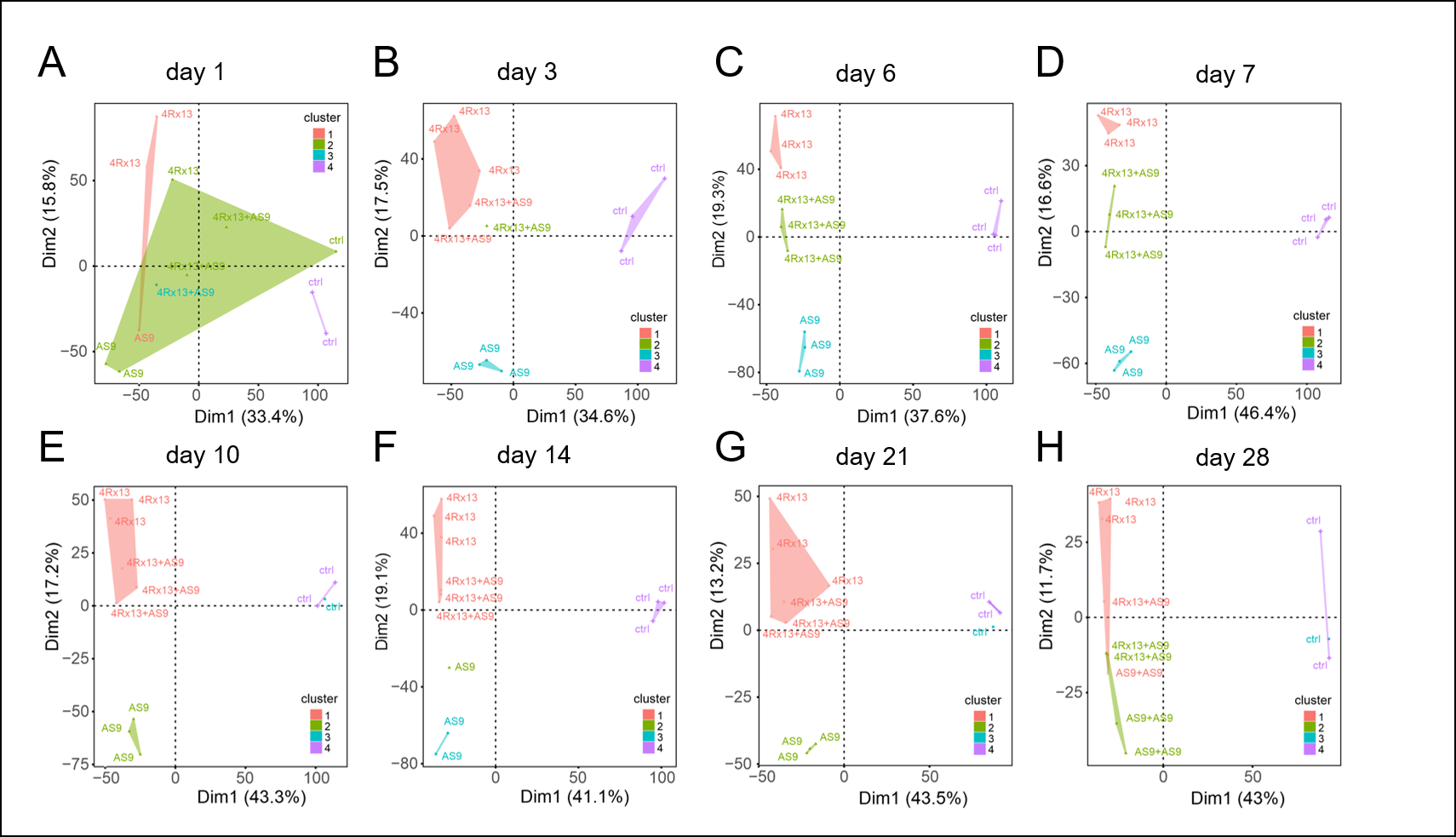

Supplement: Supplementary Figure 3 — Distinct metabolic profiles of intraspecific co-cultivations of S. plymuthica 4Rx13 and S. plymuthica B2g compared to their mono-cultures. PAM-Cluster plots. [file Image_3.TIF]

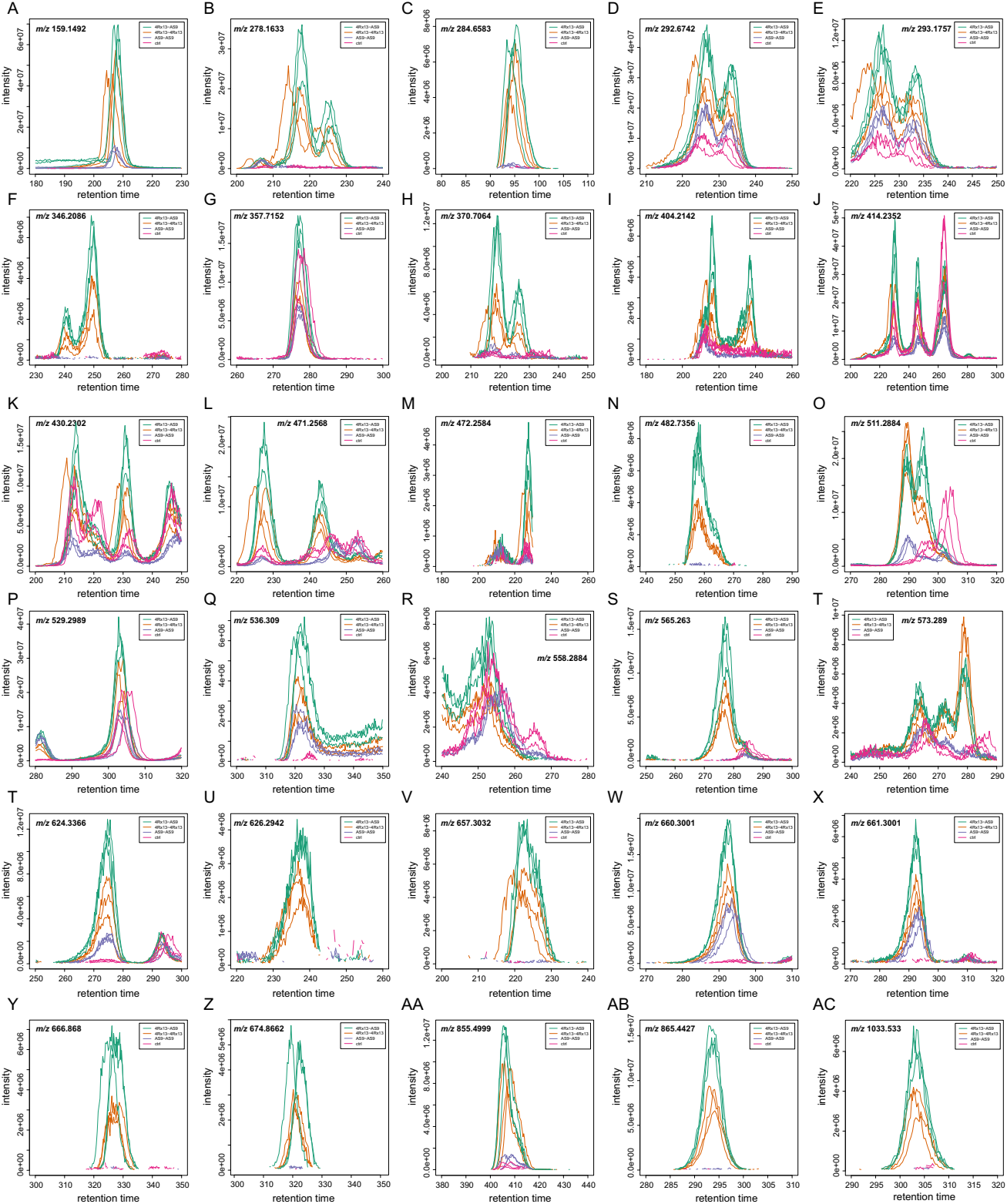

Supplement: Supplementary Figure 4 — Due to S. plymuthica 4Rx13 and AS9 co-cultivation increased features, respective m/z of every feature was extracted and plotted (cEIC). [file Image_4.pdf]
