# Supplementary material for: Metabolic Profiling of Rhizobacteria Serratia plymuthica and Bacillus subtilis Revealed Intra- and Interspecific Differences and Elicitation of Plipastatins and Short Peptides Due to Co-cultivation
Source: Front Microbiol. 2021 May 31;12:685224. doi: 10.3389/fmicb.2021.685224 (PMC8200778; doi:10.3389/fmicb.2021.685224)
Supplement: Supplementary Table 1 — Lipopeptides produced by B. subtilis B2g. [file Data_Sheet_1.zip › Supplementary Tables/Table 1.DOCX]

**Supplementary table 1**| Lipopeptides produced by *B. subtilis* B2g

| **Name** | **Chemical Formula** | **Monoisotopic mass (M) *m/z*** | **Isomers*** | **measured *m/z* [M+2H]^2+^** | **calc. *m/z* [M+2H]^2+^** | **Δppm** | **measured *m/z* [M+H]+** | **calcd. *m/z* [M+H]+** | **Δppm** |
| --- | --- | --- | --- | --- | --- | --- | --- | --- | --- |
| Surfactin C11 | C_49_H_85_N_7_O_13_ | 979.61998 | I | - | | | 980.6283 | 980.6278 | 0.5 |
| Surfactin C12 | C_50_H_87_N_7_O_13_ | 993.6356 | I | - | | | 994.6443 | 994.6434 | 0.8 |
|  |  |  | II |  |  |  | 994.6440 |  | 0.5 |
|  |  |  | III |  |  |  | 994.6443 |  | 0.8 |
| Surfactin C13 | C_51_H_89_N_7_O_13_ | 1007.6513 | I | 504.8332 | 504.8332 | 0.05 | 1008.659 | 1008.6591 | 0.1 |
|  |  |  | II | 504.833 |  | 0.5 | 1008.6596 |  | 0.5 |
|  |  |  | III | - |  | - | 1008.6592 |  | 0.1 |
|  |  |  | IV | - |  | - | 1008.6597 |  | 0.6 |
| Surfactin C14 | C_52_H_91_N_7_O_13_ | 1021.6669 | I | - | | | 1022.6751 | 1022.6748 | 0.4 |
|  |  |  | II |  |  |  | 1022.6752 |  | 0.4 |
|  |  |  | III |  |  |  | 1022.6757 |  | 0.9 |
| Surfactin C15 | C_53_H_93_N_7_O_13_ | 1035.6826 | I | 518.8491 | 518.8488 | 0.5 | 1036.6907 | 1036.6904 | 0.3 |
|  |  |  | II | 518.8494 |  | 1.1 | 1036.6915 |  | 1 |
|  |  |  | III | - |  | - | 1036.6915 |  | 1 |
| Surfactin C16 | C_54_H_95_N_7_O_13_ | 1049.6982 | I | - | | | 1050.7065 | 1050.7062 | 0.5 |
|  |  |  | II |  |  |  | 1050.7062 |  | 0.2 |
|  |  |  | III |  |  |  | 1050.7062 |  | 0.5 |
| Surfactin C17 | C_55_H_97_N_7_O_13_ | 1063.7139 | tr | - | | | 1064.7222 | 1064.7217 | 0.5 |
| Bacillomycin D1 | C_46_H_70_O_15_N_10_ | 1002.5017 |  |  |  |  | 1003.5094 | 1003.5095 | 0.1 |
| Bacillomycin D2 | C_47_H_72_O_15_N_10_ | 1016.5173 | I |  |  |  | 1017.5260 | 1017.5251 | 0.8 |
|  |  |  | II |  |  |  | 1017.5264 |  | 1.3 |
|  |  |  | III |  |  |  | 1017.5254 |  | 0.3 |
|  |  |  | IV |  |  |  | 1017.5265 |  | 1.3 |
| Bacillomycin D3 | C_48_H_74_N_10_O_15_ | 1030.533 |  |  |  |  | 1031.5423 | 1031.5407 | 1.4 |
| Bacillomycin D4 | C_49_H_76_N_10_O_15_ | 1044.5486 |  |  |  |  | 1045.5581 | 1045.5564 | 1.7 |
