# Supplementary material for: Metabolic Profiling of Rhizobacteria Serratia plymuthica and Bacillus subtilis Revealed Intra- and Interspecific Differences and Elicitation of Plipastatins and Short Peptides Due to Co-cultivation
Source: Front Microbiol. 2021 May 31;12:685224. doi: 10.3389/fmicb.2021.685224 (PMC8200778; doi:10.3389/fmicb.2021.685224)
Supplement: Supplementary Table 1 — Lipopeptides produced by B. subtilis B2g. [file Data_Sheet_1.zip › Supplementary Tables/Table 2.DOCX]

**Supplementary table 2**| Differentially induced mass features in *Serratia plymuthica* 4Rx13 interaction with *Bacillus subtilis* B2g compared to mono-cultivated strains and medium control

| **day 1** |  |  |  |  |  |  |
| --- | --- | --- | --- | --- | --- | --- |
|  | mzmed | rtmed | correlation | t-stat | p-value | FDR |
| 1 | 163.0977 | 1106.555 | 0.71871 | 4.8482 | 7.61E-05 | 0.19025 |
| 2 | 239.0636 | 161.787 | 0.64199 | 3.9274 | 0.00072 | 0.89948 |

| **day 3** |  |  |  |  |  |  |
| --- | --- | --- | --- | --- | --- | --- |
|  | mzmed | rtmed | correlation | t-stat | p-value | FDR |
| 1 | 654.3474 | 288.3258 | 0.79266 | 6.0982 | 3.88E-06 | 0.009692 |
| 2 | 553.3352 | 306.0939 | 0.70807 | 4.7032 | 0.000108 | 0.091705 |
| 3 | 523.2563 | 375.1956 | 0.69334 | 4.5129 | 0.000172 | 0.091705 |
| 4 | 463.3211 | 856.884 | 0.69188 | 4.4946 | 0.00018 | 0.091705 |
| 5 | 659.3413 | 374.0533 | 0.6882 | 4.4491 | 0.000201 | 0.091705 |
| 6 | 660.3445 | 374.1264 | 0.68521 | 4.4126 | 0.00022 | 0.091705 |
| 7 | 561.2761 | 325.3603 | 0.66127 | 4.1347 | 0.000434 | 0.1316 |
| 8 | 642.3105 | 231.2109 | 0.6595 | 4.1151 | 0.000456 | 0.1316 |
| 9 | 560.2721 | 325.3303 | 0.65802 | 4.0988 | 0.000474 | 0.1316 |
| 10 | 555.2782 | 296.973 | 0.65277 | 4.0417 | 0.000545 | 0.13616 |
| 11 | 445.3105 | 859.0085 | 0.64017 | 3.9085 | 0.000754 | 0.15594 |
| 12 | 327.211 | 301.0382 | 0.63881 | 3.8945 | 0.00078 | 0.15594 |
| 13 | 460.2643 | 359.4065 | 0.63723 | 3.8783 | 0.000811 | 0.15594 |
| 14 | 459.2609 | 359.585 | 0.62603 | 3.7655 | 0.001067 | 0.18002 |
| 15 | 588.3037 | 359.125 | 0.62184 | 3.7243 | 0.001179 | 0.18002 |
| 16 | 589.3073 | 359.1138 | 0.62076 | 3.7138 | 0.001209 | 0.18002 |
| 17 | 660.3444 | 371.4155 | 0.61784 | 3.6855 | 0.001295 | 0.18002 |
| 18 | 604.3183 | 286.1216 | 0.61603 | 3.6681 | 0.00135 | 0.18002 |
| 19 | 732.4053 | 615.642 | 0.61545 | 3.6625 | 0.001369 | 0.18002 |
| 20 | 573.2891 | 301.9633 | 0.61112 | 3.6213 | 0.001512 | 0.18255 |
| 21 | 360.1921 | 347.0804 | 0.61048 | 3.6152 | 0.001534 | 0.18255 |

| **day 6** |  |  |  |  |  |  |
| --- | --- | --- | --- | --- | --- | --- |
|  | **mzmed** | **rtmed** | **correlation** | **t-stat** | **p-value** | **FDR** |
| 1 | 750.9043 | 640.0455 | 0.70853 | 4.7094 | 0.000107 | 0.26673 |
| 2 | 747.4252 | 653.7017 | 0.65547 | 4.0709 | 0.000507 | 0.38025 |
| 3 | 733.4088 | 627.0871 | 0.63717 | 3.8776 | 0.000813 | 0.38025 |
| 4 | 553.3356 | 379.2876 | -0.61508 | -3.659 | 0.00138 | 0.38025 |
| 5 | 522.2205 | 280.1333 | -0.61168 | -3.6266 | 0.001493 | 0.38025 |
| 6 | 1031.542 | 584.6185 | 0.60103 | 3.5273 | 0.001896 | 0.38025 |

| **day 10** |  |  |  |  |  |  |
| --- | --- | --- | --- | --- | --- | --- |
|  | **mzmed** | **rtmed** | **correlation** | **t-stat** | **p-value** | **FDR** |
| 1 | 678.3106 | 311.6641 | 0.90505 | 9.9811 | 1.25E-09 | 2.41E-06 |
| 2 | 1031.541 | 584.6827 | 0.90104 | 9.7439 | 1.93E-09 | 2.41E-06 |
| 3 | 741.4109 | 591.4041 | 0.89553 | 9.439 | 3.41E-09 | 2.84E-06 |
| 4 | 399.1878 | 216.3264 | 0.85903 | 7.8708 | 7.73E-08 | 4.83E-05 |
| 5 | 489.2355 | 313.819 | 0.8416 | 7.3087 | 2.56E-07 | 0.000128 |
| 6 | 758.3868 | 638.9579 | 0.82896 | 6.9517 | 5.59E-07 | 0.000233 |
| 7 | 741.9124 | 591.5133 | 0.81624 | 6.6269 | 1.16E-06 | 0.000413 |
| 8 | 747.4252 | 652.4258 | 0.79275 | 6.1001 | 3.86E-06 | 0.001206 |
| 9 | 588.3045 | 367.8269 | 0.78473 | 5.9382 | 5.63E-06 | 0.001563 |
| 10 | 654.3472 | 283.7869 | 0.76904 | 5.6431 | 1.13E-05 | 0.002753 |
| 11 | 506.2616 | 639.3453 | 0.76734 | 5.6128 | 1.21E-05 | 0.002753 |
| 12 | 739.9159 | 658.2961 | 0.72355 | 4.9165 | 6.45E-05 | 0.013433 |
| 13 | 754.4324 | 662.3519 | 0.71631 | 4.815 | 8.25E-05 | 0.015866 |
| 14 | 415.2198 | 277.205 | 0.70436 | 4.6542 | 0.000122 | 0.020818 |
| 15 | 220.1183 | 261.9643 | 0.70161 | 4.6183 | 0.000133 | 0.020818 |
| 16 | 739.4135 | 614.4107 | 0.7016 | 4.6182 | 0.000133 | 0.020818 |
| 17 | 553.3354 | 360.7966 | -0.69565 | -4.542 | 0.000161 | 0.023599 |
| 18 | 200.1395 | 96.88852 | -0.68607 | -4.4231 | 0.000215 | 0.029797 |
| 19 | 524.309 | 323.5061 | 0.6829 | 4.3847 | 0.000236 | 0.031005 |
| 20 | 372.1922 | 358.2357 | 0.66817 | 4.2123 | 0.000359 | 0.044798 |
| 21 | 741.9122 | 588.4304 | 0.66648 | 4.1931 | 0.000376 | 0.044798 |
| 22 | 612.2997 | 240.568 | 0.65752 | 4.0933 | 0.00048 | 0.054564 |
| 23 | 758.8879 | 639.3008 | 0.65523 | 4.0682 | 0.000511 | 0.055486 |
| 24 | 613.3032 | 241.0057 | 0.64581 | 3.9674 | 0.000653 | 0.06587 |
| 25 | 512.2555 | 225.73 | 0.64545 | 3.9637 | 0.000659 | 0.06587 |
| 26 | 746.4224 | 652.7291 | 0.64256 | 3.9334 | 0.000709 | 0.068187 |
| 27 | 505.9272 | 638.1584 | 0.63987 | 3.9055 | 0.000759 | 0.070277 |
| 28 | 746.9238 | 652.7291 | 0.63702 | 3.8761 | 0.000815 | 0.072777 |
| 29 | 421.2247 | 242.5421 | 0.63548 | 3.8604 | 0.000847 | 0.073009 |
| 30 | 505.9274 | 640.7228 | 0.63249 | 3.8301 | 0.000912 | 0.07597 |
| 31 | 316.187 | 241.9491 | 0.62926 | 3.7976 | 0.000987 | 0.079551 |
| 32 | 762.434 | 622.8282 | 0.6238 | 3.7435 | 0.001125 | 0.085363 |
| 33 | 500.9357 | 638.1684 | 0.62372 | 3.7427 | 0.001127 | 0.085363 |
| 34 | 534.2668 | 616.6595 | 0.62033 | 3.7096 | 0.001222 | 0.089777 |
| 35 | 753.4299 | 675.5349 | 0.61874 | 3.6942 | 0.001268 | 0.090507 |
| 36 | 511.2519 | 225.5928 | 0.608 | 3.5919 | 0.001623 | 0.11213 |
| 37 | 753.9313 | 675.5349 | 0.60699 | 3.5825 | 0.00166 | 0.11213 |
| 38 | 596.3047 | 255.1049 | 0.60498 | 3.5637 | 0.001737 | 0.11421 |
| 39 | 543.2583 | 614.9166 | 0.60141 | 3.5307 | 0.00188 | 0.11827 |
| 40 | 279.1342 | 256.5442 | 0.6011 | 3.5279 | 0.001893 | 0.11827 |

| **day 14** |  |  |  |  |  |  |
| --- | --- | --- | --- | --- | --- | --- |
|  | **mzmed** | **rtmed** | **correlation** | **t-stat** | **p-value** | **FDR** |
| 1 | 740.4158 | 616.7712 | 0.90624 | 10.054 | 1.09E-09 | 2.43E-06 |
| 2 | 740.9181 | 616.8374 | 0.90096 | 9.7395 | 1.95E-09 | 2.43E-06 |
| 3 | 329.316 | 585.2714 | 0.88761 | 9.0387 | 7.34E-09 | 6.12E-06 |
| 4 | 742.4135 | 589.9408 | 0.83015 | 6.9838 | 5.21E-07 | 0.000326 |
| 5 | 741.9121 | 588.8329 | 0.8196 | 6.7098 | 9.59E-07 | 0.00048 |
| 6 | 758.3866 | 639.0088 | 0.81444 | 6.5836 | 1.28E-06 | 0.000531 |
| 7 | 678.3109 | 313.8107 | 0.80834 | 6.4402 | 1.77E-06 | 0.00063 |
| 8 | 755.4263 | 605.2806 | 0.78815 | 6.0062 | 4.80E-06 | 0.0015 |
| 9 | 766.3968 | 638.8887 | 0.78311 | 5.9064 | 6.06E-06 | 0.001684 |
| 10 | 755.9278 | 605.3047 | 0.76975 | 5.656 | 1.09E-05 | 0.002545 |
| 11 | 169.0358 | 204.258 | 0.76919 | 5.6459 | 1.12E-05 | 0.002545 |
| 12 | 496.2408 | 216.0159 | 0.76652 | 5.5982 | 1.25E-05 | 0.002612 |
| 13 | 399.1877 | 212.741 | 0.75914 | 5.4701 | 1.70E-05 | 0.00327 |
| 14 | 747.4251 | 652.116 | 0.75414 | 5.3862 | 2.08E-05 | 0.003639 |
| 15 | 747.9265 | 651.766 | 0.75288 | 5.3655 | 2.18E-05 | 0.003639 |
| 16 | 286.1404 | 173.6842 | 0.74279 | 5.2037 | 3.22E-05 | 0.00475 |
| 17 | 506.2616 | 639.896 | 0.74271 | 5.2024 | 3.23E-05 | 0.00475 |
| 18 | 731.417 | 651.5541 | 0.73773 | 5.1256 | 3.89E-05 | 0.0054 |
| 19 | 717.4007 | 637.206 | 0.726 | 4.9516 | 5.92E-05 | 0.007791 |
| 20 | 369.1888 | 303.8776 | 0.7165 | 4.8176 | 8.20E-05 | 0.010249 |
| 21 | 739.4135 | 617.0806 | 0.71181 | 4.7534 | 9.59E-05 | 0.011409 |
| 22 | 726.3999 | 596.1913 | 0.70829 | 4.7061 | 0.000108 | 0.011571 |
| 23 | 739.9146 | 616.8107 | 0.70782 | 4.6999 | 0.000109 | 0.011571 |
| 24 | 496.5833 | 595.1011 | 0.70622 | 4.6787 | 0.000115 | 0.011571 |
| 25 | 505.9275 | 639.3079 | 0.70602 | 4.6761 | 0.000116 | 0.011571 |
| 26 | 741.4106 | 589.8065 | 0.70182 | 4.621 | 0.000132 | 0.012725 |
| 27 | 746.9236 | 652.6279 | 0.70034 | 4.6019 | 0.000139 | 0.012838 |
| 28 | 279.1342 | 258.937 | 0.69502 | 4.5341 | 0.000164 | 0.014608 |
| 29 | 758.888 | 639.0395 | 0.67948 | 4.3438 | 0.000261 | 0.022448 |
| 30 | 497.2519 | 594.803 | 0.67767 | 4.3225 | 0.000274 | 0.022862 |
| 31 | 748.9198 | 603.7824 | 0.6754 | 4.2957 | 0.000293 | 0.02362 |
| 32 | 739.4148 | 658.3886 | 0.66051 | 4.1263 | 0.000443 | 0.034611 |
| 33 | 746.4224 | 652.7302 | 0.65272 | 4.0411 | 0.000546 | 0.040268 |
| 34 | 725.8989 | 601.1882 | 0.65257 | 4.0394 | 0.000548 | 0.040268 |
| 35 | 739.916 | 658.3256 | 0.64587 | 3.968 | 0.000652 | 0.046554 |
| 36 | 726.9014 | 596.1913 | 0.64433 | 3.9519 | 0.000678 | 0.047079 |
| 37 | 344.3349 | 612.8942 | 0.64093 | 3.9164 | 0.000739 | 0.049938 |
| 38 | 331.1657 | 252.6867 | -0.62639 | -3.769 | 0.001058 | 0.06844 |
| 39 | 343.2085 | 95.77248 | 0.62598 | 3.765 | 0.001068 | 0.06844 |
| 40 | 718.8916 | 589.6413 | 0.62376 | 3.7432 | 0.001126 | 0.070349 |
| 41 | 497.7337 | 239.0974 | 0.61649 | 3.6725 | 0.001336 | 0.081431 |
| 42 | 718.3903 | 589.7924 | 0.6147 | 3.6554 | 0.001392 | 0.081566 |
| 43 | 759.389 | 638.754 | 0.61436 | 3.6521 | 0.001404 | 0.081566 |
| 44 | 763.4371 | 620.1945 | 0.61095 | 3.6197 | 0.001518 | 0.086028 |
| 45 | 553.3359 | 378.5439 | -0.6092 | -3.6032 | 0.001579 | 0.086028 |
| 46 | 268.1404 | 208.4554 | 0.60825 | 3.5943 | 0.001614 | 0.086028 |
| 47 | 762.9353 | 620.2208 | 0.60813 | 3.5931 | 0.001618 | 0.086028 |
| 48 | 749.4212 | 603.4897 | 0.60364 | 3.5514 | 0.001789 | 0.091338 |
| 49 | 500.9356 | 640.7971 | 0.6036 | 3.551 | 0.001791 | 0.091338 |

| **day 21** |  |  |  |  |  |  |
| --- | --- | --- | --- | --- | --- | --- |
|  | **mzmed** | **rtmed** | **correlation** | **t-stat** | **p-value** | **FDR** |
| 1 | 741.4109 | 587.4069 | 0.981 | 23.943 | 3.01E-17 | 7.51E-14 |
| 2 | 748.9197 | 603.903 | 0.935 | 12.36 | 2.25E-11 | 2.81E-08 |
| 3 | 740.9175 | 615.6468 | 0.925 | 11.455 | 9.66E-11 | 8.04E-08 |
| 4 | 306.1203 | 305.3985 | 0.885 | 8.9337 | 9.01E-09 | 5.63E-06 |
| 5 | 742.4137 | 587.5162 | 0.874 | 8.437 | 2.41E-08 | 1.21E-05 |
| 6 | 741.9122 | 587.7883 | 0.866 | 8.1344 | 4.47E-08 | 1.86E-05 |
| 7 | 739.9148 | 615.8827 | 0.859 | 7.871 | 7.72E-08 | 2.76E-05 |
| 8 | 739.4138 | 615.8827 | 0.856 | 7.7558 | 9.84E-08 | 3.07E-05 |
| 9 | 732.4062 | 610.8312 | 0.835 | 7.126 | 3.81E-07 | 0.000106 |
| 10 | 732.9076 | 610.8312 | 0.813 | 6.5404 | 1.41E-06 | 0.000351 |
| 11 | 510.9338 | 651.8235 | 0.809 | 6.4506 | 1.72E-06 | 0.000392 |
| 12 | 510.5995 | 651.7764 | 0.794 | 6.1227 | 3.66E-06 | 0.000763 |
| 13 | 417.2146 | 343.786 | 0.787 | 5.9832 | 5.07E-06 | 0.000974 |
| 14 | 756.4297 | 604.5329 | 0.784 | 5.9181 | 5.90E-06 | 0.001053 |
| 15 | 733.4091 | 605.1306 | 0.779 | 5.8285 | 7.28E-06 | 0.001213 |
| 16 | 741.4099 | 598.228 | 0.777 | 5.7957 | 7.86E-06 | 0.001228 |
| 17 | 747.9264 | 651.431 | 0.770 | 5.6654 | 1.07E-05 | 0.001572 |
| 18 | 740.4153 | 616.2471 | 0.762 | 5.5108 | 1.54E-05 | 0.002144 |
| 19 | 736.3876 | 599.1235 | 0.756 | 5.4198 | 1.92E-05 | 0.002523 |
| 20 | 726.4 | 599.3954 | 0.754 | 5.3797 | 2.11E-05 | 0.002552 |
| 21 | 511.9187 | 639.3411 | 0.753 | 5.3733 | 2.14E-05 | 0.002552 |
| 22 | 744.371 | 599.1125 | 0.750 | 5.3199 | 2.44E-05 | 0.002768 |
| 23 | 717.9022 | 636.8362 | 0.739 | 5.1484 | 3.68E-05 | 0.004 |
| 24 | 678.3111 | 315.8026 | 0.733 | 5.0551 | 4.61E-05 | 0.004611 |
| 25 | 763.4372 | 619.9499 | 0.733 | 5.0549 | 4.61E-05 | 0.004611 |
| 26 | 762.9355 | 620.0257 | 0.731 | 5.0218 | 5.00E-05 | 0.004804 |
| 27 | 681.3582 | 268.221 | 0.727 | 4.9731 | 5.62E-05 | 0.005072 |
| 28 | 762.4342 | 620.0795 | 0.727 | 4.9688 | 5.68E-05 | 0.005072 |
| 29 | 718.3901 | 586.9576 | 0.712 | 4.7624 | 9.38E-05 | 0.007989 |
| 30 | 739.4138 | 612.4276 | 0.712 | 4.7533 | 9.59E-05 | 0.007989 |
| 31 | 734.904 | 580.3564 | 0.709 | 4.7188 | 0.000104 | 0.008409 |
| 32 | 415.2198 | 278.5631 | 0.707 | 4.6871 | 0.000113 | 0.0088 |
| 33 | 747.4251 | 651.694 | 0.706 | 4.6701 | 0.000117 | 0.008894 |
| 34 | 746.9237 | 652.1147 | 0.698 | 4.5769 | 0.000147 | 0.010603 |
| 35 | 746.4225 | 652.0072 | 0.698 | 4.5739 | 0.000149 | 0.010603 |
| 36 | 725.398 | 599.3954 | 0.695 | 4.5312 | 0.000165 | 0.011385 |
| 37 | 753.4297 | 653.2853 | 0.694 | 4.522 | 0.000169 | 0.011385 |
| 38 | 656.3266 | 271.5271 | 0.688 | 4.4452 | 0.000203 | 0.013374 |
| 39 | 500.2268 | 362.4466 | 0.687 | 4.4295 | 0.000211 | 0.013539 |
| 40 | 731.9186 | 651.1327 | 0.679 | 4.3377 | 0.000264 | 0.016521 |
| 41 | 274.1193 | 372.3727 | 0.676 | 4.3071 | 0.000285 | 0.017123 |
| 42 | 726.9015 | 599.3849 | 0.676 | 4.3031 | 0.000288 | 0.017123 |
| 43 | 749.421 | 603.7454 | 0.671 | 4.2492 | 0.000328 | 0.019013 |
| 44 | 717.4007 | 636.7115 | 0.670 | 4.2347 | 0.00034 | 0.019013 |
| 45 | 678.3114 | 312.6873 | 0.670 | 4.232 | 0.000342 | 0.019013 |
| 46 | 755.4263 | 604.673 | 0.667 | 4.1975 | 0.000372 | 0.020234 |
| 47 | 734.9053 | 561.6003 | 0.666 | 4.1856 | 0.000383 | 0.02039 |
| 48 | 755.9277 | 604.6212 | 0.665 | 4.172 | 0.000396 | 0.020638 |
| 49 | 221.1216 | 260.6905 | 0.661 | 4.1307 | 0.000438 | 0.021974 |
| 50 | 510.5989 | 637.7893 | 0.661 | 4.1296 | 0.00044 | 0.021974 |
| 51 | 758.3869 | 638.9173 | 0.659 | 4.1141 | 0.000457 | 0.022146 |
| 52 | 261.1068 | 339.8388 | 0.659 | 4.1052 | 0.000467 | 0.022146 |
| 53 | 725.8989 | 599.3954 | 0.658 | 4.0995 | 0.000473 | 0.022146 |
| 54 | 222.0797 | 218.5755 | 0.658 | 4.0949 | 0.000479 | 0.022146 |
| 55 | 496.9173 | 599.099 | 0.657 | 4.0834 | 0.000492 | 0.022363 |
| 56 | 750.4027 | 638.7658 | 0.651 | 4.022 | 0.000572 | 0.025507 |
| 57 | 506.2614 | 638.786 | 0.648 | 3.9948 | 0.000611 | 0.02678 |
| 58 | 242.1004 | 264.8082 | 0.641 | 3.9189 | 0.000735 | 0.031663 |
| 59 | 357.3478 | 648.8927 | 0.640 | 3.9016 | 0.000766 | 0.032137 |
| 60 | 388.1875 | 304.6331 | 0.639 | 3.8988 | 0.000772 | 0.032137 |
| 61 | 732.4062 | 605.1306 | 0.637 | 3.8763 | 0.000815 | 0.033392 |
| 62 | 496.5831 | 599.3849 | 0.636 | 3.8683 | 0.000831 | 0.033503 |
| 63 | 1031.541 | 579.0911 | 0.630 | 3.8099 | 0.000958 | 0.037997 |
| 64 | 731.4171 | 651.1327 | 0.627 | 3.779 | 0.001032 | 0.040312 |
| 65 | 535.2479 | 579.0911 | 0.626 | 3.764 | 0.001071 | 0.041161 |
| 66 | 343.3317 | 613.1218 | 0.620 | 3.7075 | 0.001228 | 0.046476 |
| 67 | 541.2627 | 220.0833 | 0.615 | 3.654 | 0.001397 | 0.052114 |
| 68 | 534.2669 | 614.64 | 0.609 | 3.5992 | 0.001595 | 0.058058 |
| 69 | 1450.791 | 599.4266 | 0.608 | 3.592 | 0.001623 | 0.058058 |
| 70 | 175.1083 | 343.5803 | 0.607 | 3.5823 | 0.001661 | 0.058058 |
| 71 | 573.2524 | 218.8831 | 0.607 | 3.5801 | 0.00167 | 0.058058 |
| 72 | 372.1924 | 363.8571 | 0.607 | 3.5793 | 0.001673 | 0.058058 |

| **day 28** |  |  |  |  |  |  |
| --- | --- | --- | --- | --- | --- | --- |
|  | **mzmed** | **rtmed** | **correlation** | **t-stat** | **p-value** | **FDR** |
| 1 | 740.9181 | 703.0642 | 0.9103 | 10.314 | 6.85E-10 | 1.71E-06 |
| 2 | 748.9205 | 690.673 | 0.90371 | 9.9 | 1.45E-09 | 1.81E-06 |
| 3 | 204.087 | 290.2277 | 0.89108 | 9.2093 | 5.29E-09 | 4.40E-06 |
| 4 | 204.087 | 291.384 | 0.8751 | 8.4817 | 2.21E-08 | 1.38E-05 |
| 5 | 204.087 | 316.6707 | 0.86896 | 8.2357 | 3.63E-08 | 1.82E-05 |
| 6 | 740.4154 | 703.1582 | 0.85355 | 7.6842 | 1.14E-07 | 4.77E-05 |
| 7 | 567.3905 | 153.8005 | 0.84403 | 7.3818 | 2.18E-07 | 7.80E-05 |
| 8 | 343.332 | 727.051 | 0.82834 | 6.9352 | 5.80E-07 | 0.000181 |
| 9 | 741.4113 | 673.6533 | 0.80615 | 6.3902 | 1.98E-06 | 0.00055 |
| 10 | 741.9131 | 658.5819 | 0.80379 | 6.3372 | 2.23E-06 | 0.000559 |
| 11 | 734.4038 | 642.6594 | 0.7913 | 6.0703 | 4.14E-06 | 0.00094 |
| 12 | 733.409 | 680.0895 | 0.78096 | 5.8648 | 6.69E-06 | 0.001299 |
| 13 | 748.419 | 690.345 | 0.78073 | 5.8604 | 6.76E-06 | 0.001299 |
| 14 | 388.1876 | 377.2744 | 0.77438 | 5.7406 | 8.96E-06 | 0.001599 |
| 15 | 734.4038 | 645.6678 | 0.75473 | 5.3961 | 2.03E-05 | 0.003077 |
| 16 | 612.3002 | 303.507 | 0.75343 | 5.3745 | 2.14E-05 | 0.003077 |
| 17 | 748.9203 | 663.3578 | 0.7527 | 5.3625 | 2.20E-05 | 0.003077 |
| 18 | 735.4066 | 641.882 | 0.75229 | 5.3558 | 2.24E-05 | 0.003077 |
| 19 | 357.3479 | 738.2364 | 0.75114 | 5.3369 | 2.34E-05 | 0.003077 |
| 20 | 613.3038 | 301.0589 | 0.74442 | 5.2293 | 3.03E-05 | 0.003785 |
| 21 | 739.915 | 702.8571 | 0.74159 | 5.185 | 3.37E-05 | 0.00401 |
| 22 | 742.4138 | 673.511 | 0.73426 | 5.0731 | 4.41E-05 | 0.004702 |
| 23 | 496.241 | 284.4359 | 0.73391 | 5.068 | 4.47E-05 | 0.004702 |
| 24 | 497.7343 | 310.1035 | 0.73363 | 5.0637 | 4.52E-05 | 0.004702 |
| 25 | 734.9053 | 642.6356 | 0.72667 | 4.9613 | 5.79E-05 | 0.005761 |
| 26 | 739.4138 | 703.135 | 0.72566 | 4.9468 | 5.99E-05 | 0.005761 |
| 27 | 230.0462 | 265.7233 | 0.71931 | 4.8566 | 7.46E-05 | 0.006904 |
| 28 | 498.2358 | 306.4193 | 0.71579 | 4.8078 | 8.40E-05 | 0.007497 |
| 29 | 733.4091 | 698.078 | 0.7142 | 4.786 | 8.86E-05 | 0.007633 |
| 30 | 678.3113 | 384.2245 | 0.7123 | 4.7601 | 9.43E-05 | 0.007701 |
| 31 | 481.2863 | 397.5466 | 0.71192 | 4.7549 | 9.55E-05 | 0.007701 |
| 32 | 656.3268 | 346.7382 | 0.7104 | 4.7344 | 0.0001 | 0.007842 |
| 33 | 222.0797 | 281.5317 | 0.70808 | 4.7033 | 0.000108 | 0.007968 |
| 34 | 387.3219 | 691.6956 | 0.70783 | 4.7001 | 0.000109 | 0.007968 |
| 35 | 732.9077 | 698.3434 | 0.70716 | 4.6911 | 0.000112 | 0.007968 |
| 36 | 206.0851 | 439.5274 | 0.70096 | 4.6099 | 0.000136 | 0.009442 |
| 37 | 388.1878 | 374.6872 | 0.69577 | 4.5435 | 0.00016 | 0.010802 |
| 38 | 498.2357 | 312.0368 | 0.69369 | 4.5173 | 0.000171 | 0.011214 |
| 39 | 678.3116 | 386.9953 | 0.68998 | 4.4711 | 0.000191 | 0.01223 |
| 40 | 741.9123 | 673.4797 | 0.68815 | 4.4485 | 0.000202 | 0.012602 |
| 41 | 750.3182 | 302.626 | 0.6863 | 4.4259 | 0.000213 | 0.012994 |
| 42 | 274.1193 | 445.5084 | 0.68354 | 4.3924 | 0.000231 | 0.013766 |
| 43 | 353.1745 | 282.6458 | 0.682 | 4.3739 | 0.000242 | 0.014065 |
| 44 | 372.1928 | 441.5744 | 0.67734 | 4.3186 | 0.000277 | 0.015737 |
| 45 | 510.9341 | 741.2597 | 0.67242 | 4.2611 | 0.000319 | 0.017634 |
| 46 | 510.6 | 741.3248 | 0.67076 | 4.242 | 0.000334 | 0.017634 |
| 47 | 372.1926 | 437.2755 | 0.67056 | 4.2396 | 0.000336 | 0.017634 |
| 48 | 1043.303 | 150.1141 | 0.67028 | 4.2364 | 0.000339 | 0.017634 |
| 49 | 747.9266 | 741.3156 | 0.6691 | 4.2229 | 0.00035 | 0.017852 |
| 50 | 763.4375 | 707.9306 | 0.66708 | 4.2 | 0.00037 | 0.018504 |
| 51 | 554.2663 | 293.402 | 0.66563 | 4.1835 | 0.000385 | 0.018887 |
| 52 | 480.2828 | 397.5466 | 0.66419 | 4.1673 | 0.000401 | 0.019001 |
| 53 | 734.9052 | 646.2523 | 0.66397 | 4.1648 | 0.000403 | 0.019001 |
| 54 | 612.3002 | 309.5474 | 0.66332 | 4.1576 | 0.000411 | 0.019001 |
| 55 | 497.7343 | 306.4193 | 0.65829 | 4.1017 | 0.000471 | 0.021383 |
| 56 | 762.9358 | 707.9421 | 0.65534 | 4.0695 | 0.000509 | 0.022719 |
| 57 | 734.4028 | 665.6405 | 0.65362 | 4.0508 | 0.000533 | 0.023096 |
| 58 | 455.1878 | 265.0921 | 0.65339 | 4.0484 | 0.000536 | 0.023096 |
| 59 | 357.3478 | 740.0252 | 0.64948 | 4.0064 | 0.000594 | 0.025154 |
| 60 | 481.2862 | 399.7673 | 0.64521 | 3.9611 | 0.000663 | 0.027351 |
| 61 | 725.3984 | 685.87 | 0.64494 | 3.9583 | 0.000668 | 0.027351 |
| 62 | 681.3584 | 343.4823 | 0.64172 | 3.9246 | 0.000725 | 0.029213 |
| 63 | 726.9021 | 685.8566 | 0.63988 | 3.9055 | 0.000759 | 0.030116 |
| 64 | 159.1494 | 278.6971 | 0.63709 | 3.8768 | 0.000814 | 0.031758 |
| 65 | 541.3445 | 413.8041 | 0.6365 | 3.8708 | 0.000826 | 0.031758 |
| 66 | 725.8994 | 685.87 | 0.63242 | 3.8293 | 0.000914 | 0.034596 |
| 67 | 839.3405 | 150.2846 | 0.63121 | 3.8171 | 0.000941 | 0.035102 |
| 68 | 762.4344 | 708.0702 | 0.6294 | 3.7991 | 0.000983 | 0.036137 |
| 69 | 741.9124 | 684.6606 | 0.62844 | 3.7894 | 0.001007 | 0.036458 |
| 70 | 373.1958 | 437.4985 | 0.62372 | 3.7427 | 0.001127 | 0.039749 |
| 71 | 487.2154 | 282.5336 | 0.62364 | 3.742 | 0.001129 | 0.039749 |
| 72 | 555.2787 | 297.9653 | 0.62304 | 3.7361 | 0.001146 | 0.039761 |
| 73 | 744.3715 | 685.8564 | 0.61927 | 3.6993 | 0.001252 | 0.042862 |
| 74 | 726.4006 | 685.8566 | 0.61801 | 3.6872 | 0.001289 | 0.043003 |
| 75 | 748.4187 | 667.6184 | 0.61798 | 3.6868 | 0.001291 | 0.043003 |
| 76 | 756.4301 | 691.2676 | 0.61725 | 3.6798 | 0.001313 | 0.043157 |
| 77 | 734.9044 | 665.9539 | 0.61297 | 3.6389 | 0.001449 | 0.047031 |
| 78 | 611.2874 | 289.5774 | 0.61198 | 3.6295 | 0.001482 | 0.047492 |
| 79 | 755.4266 | 691.2676 | 0.6114 | 3.6239 | 0.001502 | 0.047519 |
| 80 | 736.3883 | 685.8546 | 0.61038 | 3.6143 | 0.001537 | 0.048026 |
| 81 | 496.9177 | 685.8439 | 0.60566 | 3.5701 | 0.00171 | 0.052768 |
| 82 | 974.8138 | 151.0367 | 0.60389 | 3.5537 | 0.001779 | 0.054225 |
| 83 | 732.4063 | 698.078 | 0.60328 | 3.548 | 0.001804 | 0.054306 |
| 84 | 261.1241 | 391.777 | -0.60011 | -3.5188 | 0.001935 | 0.057561 |
