# Supplementary material for: Metabolic Profiling of Rhizobacteria Serratia plymuthica and Bacillus subtilis Revealed Intra- and Interspecific Differences and Elicitation of Plipastatins and Short Peptides Due to Co-cultivation
Source: Front Microbiol. 2021 May 31;12:685224. doi: 10.3389/fmicb.2021.685224 (PMC8200778; doi:10.3389/fmicb.2021.685224)
Supplement: Supplementary Table 1 — Lipopeptides produced by B. subtilis B2g. [file Data_Sheet_1.zip › Supplementary Tables/Table 3.DOCX]

**Supplementary table 3**| Time points of detection of differentially induced mass features (*m/z*) in *Serratia plymuthica* 4Rx13 interaction with *Bacillus subtilis* B2g compared to mono-cultivated strains and medium control

| **day** | **1** | **3** | **6** | **10** | **14** | **21** | **28** |
| --- | --- | --- | --- | --- | --- | --- | --- |
| 1 |  |  |  |  |  |  | 159.1494 |
| 2 | 163.0977 |  |  |  |  |  |  |
| 3 |  |  |  |  | 169.0358 |  |  |
| 4 |  |  |  |  |  | 175.1083 |  |
| 5 |  |  |  | 200.1395 |  |  |  |
| 6 |  |  |  |  |  |  | 204.087 |
| 7 |  |  |  |  |  |  | 204.087 |
| 8 |  |  |  |  |  |  | 204.087 |
| 9 |  |  |  |  |  |  | 206.0851 |
| 10 |  |  |  |  |  | 221.1216 |  |
| 11 |  |  |  |  |  | 222.0797 | 222.0797 |
| 12 |  |  |  | 220.1183 |  |  |  |
| 13 | 239.0636 |  |  |  |  |  |  |
| 14 |  |  |  |  |  |  | 230.0462 |
| 15 |  |  |  |  |  | 242.1004 |  |
| 16 |  |  |  |  |  | 261.1068 |  |
| 17 |  |  |  |  |  |  | 261.1241 |
| 18 |  |  |  |  | 268.1404 |  |  |
| 19 |  |  |  |  |  | 274.1193 | 274.1193 |
| 20 |  |  |  | 279.1342 | 279.1342 |  |  |
| 21 |  |  |  |  | 286.1404 |  |  |
| 22 |  |  |  |  |  | 306.1203 |  |
| 23 |  |  |  | 316.187 |  |  |  |
| 24 |  | 327.211 |  |  |  |  |  |
| 25 |  |  |  |  | 329.316 |  |  |
| 26 |  |  |  |  | 331.1657 |  |  |
| 27 |  |  |  |  | 343.2085 |  |  |
| 28 |  |  |  |  |  | 343.3317 | 343.332 |
| 29 |  |  |  |  | 344.3349 |  |  |
| 30 |  |  |  |  |  |  | 353.1745 |
| 31 |  |  |  |  |  |  | 357.3478 |
| 32 |  |  |  |  |  | 357.3478 | 357.3479 |
| 33 |  | 360.1921 |  |  |  |  |  |
| 34 |  |  |  |  | 369.1888 |  |  |
| 35 |  |  |  |  |  |  | 372.1926 |
| 36 |  |  |  | 372.1922 |  | 372.1924 | 372.1928 |
| 37 |  |  |  |  |  |  | 373.1958 |
| 38 |  |  |  |  |  |  | 387.3219 |
| 39 |  |  |  |  |  | 388.1875 | 388.1876 |
| 40 |  |  |  |  |  |  | 388.1878 |
| 41 |  |  |  | 399.1878 | 399.1877 |  |  |
| 42 |  |  |  | 415.2198 |  | 415.2198 |  |
| 43 |  |  |  |  | 417.2146 |  |  |
| 44 |  |  |  | 421.2247 |  |  |  |
| 45 |  | 445.3105 |  |  |  |  |  |
| 46 |  |  |  |  |  |  | 455.1878 |
| 47 |  | 459.2609 |  |  |  |  |  |
| 48 |  | 460.2643 |  |  |  |  |  |
| 49 |  | 463.3211 |  |  |  |  |  |
| 50 |  |  |  |  |  |  | 480.2828 |
| 51 |  |  |  |  |  |  | 481.2862 |
| 52 |  |  |  |  |  |  | 481.2863 |
| 53 |  |  |  |  |  |  | 487.2154 |
| 54 |  |  |  | 489.2355 |  |  |  |
| 55 |  |  |  |  | 496.2408 |  | 496.241 |
| 56 |  |  |  |  | 496.5833 | 496.5831 |  |
| 57 |  |  |  |  |  | 496.9173 | 496.9177 |
| 58 |  |  |  |  | 497.2519 |  |  |
| 59 |  |  |  |  |  |  | 497.7343 |
| 60 |  |  |  |  | 497.7337 |  | 497.7343 |
| 61 |  |  |  |  |  |  | 498.2357 |
| 62 |  |  |  |  |  |  | 498.2358 |
| 63 |  |  |  |  |  | 500.2268 |  |
| 64 |  |  |  | 500.9357 | 500.9356 |  |  |
| 65 |  |  |  | 505.9272 | 505.9275 |  |  |
| 66 |  |  |  | 505.9274 |  |  |  |
| 67 |  |  |  | 506.2616 | 506.2616 | 506.2614 |  |
| 68 |  |  |  |  |  | 510.5989 |  |
| 69 |  |  |  |  | 510.5995 |  | 510.6 |
| 70 |  |  |  |  | 510.9338 |  | 510.9341 |
| 71 |  |  |  | 511.2519 |  |  |  |
| 72 |  |  |  |  | 511.9187 |  |  |
| 73 |  |  |  | 512.2555 |  |  |  |
| 74 |  |  | 522.2205 |  |  |  |  |
| 75 |  | 523.2563 |  |  |  |  |  |
| 76 |  |  |  | 524.309 |  |  |  |
| 77 |  |  |  | 534.2668 |  | 534.2669 |  |
| 78 |  |  |  |  |  | 541.2627 |  |
| 79 |  |  |  | 553.3354 | 553.3359 |  |  |
| 80 |  |  |  |  |  | 535.2479 |  |
| 81 |  |  |  |  |  |  | 541.3445 |
| 82 |  | 553.3352 | 553.3356 | 543.2583 |  |  |  |
| 83 |  |  |  |  |  |  | 554.2663 |
| 84 |  | 555.2782 |  |  |  |  | 555.2787 |
| 85 |  | 560.2721 |  |  |  |  |  |
| 86 |  | 561.2761 |  |  |  |  |  |
| 87 |  | 573.2891 |  |  |  |  |  |
| 88 |  | 588.3037 |  | 588.3045 |  |  |  |
| 89 |  | 589.3073 |  |  |  |  |  |
| 90 |  |  |  |  |  |  | 656.3268 |
| 91 |  |  |  |  |  |  | 567.3905 |
| 92 |  |  |  |  |  | 573.2524 |  |
| 93 |  |  |  | 596.3047 |  |  |  |
| 94 |  | 604.3183 |  |  |  |  |  |
| 95 |  |  |  |  |  |  | 611.2874 |
| 96 |  |  |  |  |  |  | 612.3002 |
| 97 |  |  |  | 612.2997 |  |  | 612.3002 |
| 98 |  |  |  | 613.3032 |  |  | 613.3038 |
| 99 |  | 642.3105 |  |  |  |  |  |
| 100 |  | 654.3474 |  | 654.3472 |  |  |  |
| 101 |  |  |  |  |  | 656.3266 |  |
| 102 |  | 659.3413 |  |  |  |  |  |
| 103 |  | 660.3444 |  |  |  |  |  |
| 104 |  | 660.3445 |  |  |  |  |  |
| 105 |  |  |  | 678.3106 | 678.3109 | 678.3111 | 678.3113 |
| 106 |  |  |  |  |  | 678.3114 | 678.3116 |
| 107 |  |  |  |  | 681.3582 |  | 681.3584 |
| 108 |  |  |  |  | 717.4007 | 717.4007 |  |
| 109 |  |  |  |  |  | 717.9022 |  |
| 110 |  |  |  |  | 718.3903 | 718.3901 |  |
| 111 |  |  |  |  | 718.8916 |  |  |
| 112 |  |  |  |  |  | 725.398 | 725.3984 |
| 113 |  |  |  |  | 725.8989 | 725.8989 | 725.8994 |
| 114 |  |  |  |  | 726.3999 | 726.4 | 726.4006 |
| 115 |  |  |  |  | 726.9014 | 726.9015 | 726.9021 |
| 116 |  |  |  |  | 731.417 | 731.4171 |  |
| 117 |  |  |  |  |  | 731.9186 |  |
| 118 |  | 732.4053 |  |  |  | 732.4062 |  |
| 119 |  |  |  |  |  | 732.4062 | 732.4063 |
| 120 |  |  |  |  |  | 732.9076 | 732.9077 |
| 121 |  |  | 733.4088 |  |  | 733.4091 | 733.409 |
| 122 |  |  |  |  |  |  | 733.4091 |
| 123 |  |  |  |  |  |  | 734.4028 |
| 124 |  |  |  |  |  |  | 734.4038 |
| 125 |  |  |  |  |  |  | 734.4038 |
| 126 |  |  |  |  |  | 734.904 | 734.9044 |
| 127 |  |  |  |  |  |  | 734.9052 |
| 128 |  |  |  |  |  | 734.9053 | 734.9053 |
| 129 |  |  |  |  |  |  | 735.4066 |
| 130 |  |  |  |  |  | 736.3876 | 736.3883 |
| 131 |  |  |  | 739.4135 | 739.4135 | 739.4138 | 739.4138 |
| 132 |  |  |  |  | 739.4148 | 739.4138 |  |
| 133 |  |  |  | 739.9159 | 739.9146 | 739.9148 | 739.915 |
| 134 |  |  |  |  | 739.916 |  |  |
| 135 |  |  |  |  | 740.4158 | 740.4153 | 740.4154 |
| 136 |  |  |  |  | 740.9181 | 740.9175 | 740.9181 |
| 137 |  |  |  |  |  | 741.4099 |  |
| 138 |  |  |  | 741.4109 | 741.4106 | 741.4109 | 741.4113 |
| 139 |  |  |  | 741.9124 | 741.9121 | 741.9122 | 741.9123 |
| 140 |  |  |  | 741.9122 |  |  | 741.9124 |
| 141 |  |  |  |  |  |  | 741.9131 |
| 142 |  |  |  |  | 742.4135 | 742.4137 | 742.4138 |
| 143 |  |  |  |  |  | 744.371 | 744.3715 |
| 144 |  |  |  | 746.4224 | 746.4224 | 746.4225 |  |
| 145 |  |  |  | 746.9238 | 746.9236 | 746.9237 |  |
| 146 |  |  | 747.4252 | 747.4252 | 747.4251 | 747.4251 |  |
| 147 |  |  |  |  | 747.9265 | 747.9264 | 747.9266 |
| 148 |  |  |  |  |  |  | 748.4187 |
| 149 |  |  |  |  |  | 748.4182 | 748.419 |
| 150 |  |  |  |  |  |  | 748.9203 |
| 151 |  |  |  |  | 748.9198 | 748.9197 | 748.9205 |
| 152 |  |  |  |  | 749.4212 | 749.421 |  |
| 153 |  |  |  |  |  |  | 750.3182 |
| 154 |  |  |  |  |  | 750.4027 |  |
| 155 |  |  | 750.9043 |  |  |  |  |
| 156 |  |  |  | 753.4299 |  | 753.4297 |  |
| 157 |  |  |  | 753.9313 |  |  |  |
| 158 |  |  |  | 754.4324 |  |  |  |
| 159 |  |  |  |  | 755.4263 | 755.4263 | 755.4266 |
| 160 |  |  |  |  | 755.9278 | 755.9277 |  |
| 161 |  |  |  |  | 756.4297 |  | 756.4301 |
| 162 |  |  |  | 758.3868 | 758.3866 | 758.3869 |  |
| 163 |  |  |  | 758.8879 | 758.888 |  |  |
| 164 |  |  |  |  | 759.389 |  |  |
| 165 |  |  |  | 762.434 |  | 762.4342 | 762.4344 |
| 166 |  |  |  |  | 762.9353 | 762.9355 | 762.9358 |
| 167 |  |  |  |  | 763.4371 | 763.4372 | 763.4375 |
| 168 |  |  |  |  | 766.3968 |  |  |
| 169 |  |  |  |  |  |  | 839.3405 |
| 170 |  |  |  |  |  |  | 974.8138 |
| 171 |  |  | 1031.542 | 1031.541 |  | 1031.541 |  |
| 172 |  |  |  |  |  |  | 1043.303 |
| 173 |  |  |  |  |  | 1450.791 |  |
