# Supplementary material for: Metabolic Profiling of Rhizobacteria Serratia plymuthica and Bacillus subtilis Revealed Intra- and Interspecific Differences and Elicitation of Plipastatins and Short Peptides Due to Co-cultivation
Source: Front Microbiol. 2021 May 31;12:685224. doi: 10.3389/fmicb.2021.685224 (PMC8200778; doi:10.3389/fmicb.2021.685224)
Supplement: Supplementary Table 1 — Lipopeptides produced by B. subtilis B2g. [file Data_Sheet_1.zip › Supplementary Tables/Table 4.DOCX]

| ***m/z* [M+H]^+^** | **Isomer** | **plipastatin** | **Measured marker fragment *m/z*** | **Marker for aa at 6 /10 in the peptide ring** | **Chemical formula** | **Calculated m/z** | **ppm** |
| --- | --- | --- | --- | --- | --- | --- | --- |
| 1449.7898 | I | A1 | 966.45941 | Alanine/Isoleucine | C_46_H_64_N_9_O_14_ | 966.45672 | 2.8 |
|  |  |  | 1080.53918 |  | C_51_H_74_N_11_O_15_ | 1080.53603 | 2.9 |
|  | II | A1 | 966.45477 | Alanine/Isoleucine | C_46_H_64_N_9_O_14_ | 966.45672 | 2 |
|  |  |  | 1080.53943 |  | C_51_H_74_N_11_O_15_ | 1080.53603 | 3.1 |
|  | III | A2 | 1066.51892 | Alanine/Valine | C_50_H_72_N_11_O_15_ | 1066.52038 | 1.4 |
| 1463.8047 | I | B1 | 994.49011 | Valine/Isoleucine | C_48_H_68_N_9_O_14_ | 994.48802 | 2 |
|  |  |  | 1108.56982 |  | C_53_H_78_N_11_O_15_ | 1108.56733 | 2.2 |
|  | II | B2 | 980.47375 | Valine/Valine | C_47_H_66_N_9_O_14_ | 980.47237 | 1.4 |
|  |  |  | 1094.55078 |  | C_52_H_76_N_11_O_15_ | 1094.55169 | 0.8 |
|  | III | *A1* | 966.45782 | Alanine/Isoleucine | C_46_H_64_N_9_O_14_ | 966.45672 | 1.1 |
|  |  |  | 1080.53516 |  | C_51_H_74_N_11_O_15_ | 1080.53603 | 0.8 |
|  |  | B2 | 980.47394 | Valine/Valine | C_47_H_66_N_9_O_14_ | 980.47237 | 1.6 |
|  |  |  | 1094.55005 |  | C_52_H_76_N_11_O_15_ | 1094.55169 | 1.5 |
|  | IV | A1 | 966.45782 | Alanine/Isoleucine | C_46_H_64_N_9_O_14_ | 966.45672 | 1.1 |
|  |  |  | 1080.53772 |  | C_51_H_74_N_11_O_15_ | 1080.53603 | 1.6 |
| 1477.8208 | I | B1 | 994.49005 | Valine/Isoleucine | C_48_H_68_N_9_O_14_ | 994.48802 | 1.5 |
|  |  |  | 1108.5698 |  | C_53_H_78_N_11_O_15_ | 1108.56733 | 1.7 |
|  | II | B1 | 994.48871 | Valine/Isoleucine | C_48_H_68_N_9_O_14_ | 994.48802 | 0.7 |
|  |  |  | 1108.56421 |  | C_53_H_78_N_11_O_15_ | 1108.56733 | 2 |
|  | III | B2 | 980.46960 | Valine/Valine | C_47_H_66_N_9_O_14_ | 980.47237 | 2.9 |
|  |  |  | 1094.54919 |  | C_52_H_76_N_11_O_15_ | 1094.55169 | 2.3 |
|  | IV | A1 | 966.45782 | Alanine/Isoleucine | C_46_H_64_N_9_O_14_ | 966.45672 | 0.8 |
|  |  |  | 1080.53748 |  | C_51_H_74_N_11_O_15_ | 1080.53603 | 0.8 |
| 1491.8362 | I | B1 | 994.48767 | Valine/Isoleucine | C_48_H_68_N_9_O_14_ | 994.48802 | 0.4 |
|  |  |  | 1108.56726 |  | C_53_H_78_N_11_O_15_ | 1108.56733 | 0.069 |
|  | II | B2 | 980.47272 | Valine/Valine | C_47_H_66_N_9_O_14_ | 980.47237 | 0.4 |
|  |  |  | 1094.54907 |  | C_52_H_76_N_11_O_15_ | 1094.55169 | 2.4 |
|  | III | A1 | 966.45709 | Alanine/Isoleucine | C_46_H_64_N_9_O_14_ | 966.45672 | 0.4 |
|  |  |  | 1080.53589 |  | C_51_H_74_N_11_O_15_ | 1080.53603 | 0.1 |

**Supplementary table 4**| Identification of Plipastatin isomers (isomers that are more pronounced in interaction are labeled in red
