# Supplementary material for: Metabolic Profiling of Rhizobacteria Serratia plymuthica and Bacillus subtilis Revealed Intra- and Interspecific Differences and Elicitation of Plipastatins and Short Peptides Due to Co-cultivation
Source: Front Microbiol. 2021 May 31;12:685224. doi: 10.3389/fmicb.2021.685224 (PMC8200778; doi:10.3389/fmicb.2021.685224)
Supplement: Supplementary Table 1 — Lipopeptides produced by B. subtilis B2g. [file Data_Sheet_1.zip › Supplementary Tables/Table 5.DOCX]

| **day 1** |  |  |  |  |  |  |
| --- | --- | --- | --- | --- | --- | --- |
|  | mzmed | rtmed | correlation | t-stat | p-value | FDR |
| 1 | 359.2297 | 299.0396 | -0.90435 | -6.7006 | 5.36E-05 | 0.13397 |
| 2 | 233.0638 | 136.7601 | 0.81539 | 4.454 | 0.001227 | 0.99994 |

**Supplementary table 5**| Differentially induced mass features in *Serratia plymuthica* 4Rx13 interaction with *S. plymuthica* AS9 compared to mono-cultivated strains and medium control

| **day 3** |  |  |  |  |  |  |
| --- | --- | --- | --- | --- | --- | --- |
|  | mzmed | rtmed | correlation | t-stat | p-value | FDR |
| 1 | 541.344 | 342.473 | 0.8798 | 5.8529 | 0.00016 | 0.18483 |
| 2 | 525.314 | 285.133 | 0.87503 | 5.7162 | 0.00019 | 0.18483 |
| 3 | 427.257 | 274.142 | 0.87146 | 5.6186 | 0.00022 | 0.18483 |
| 4 | 563.324 | 416.814 | 0.83923 | 4.8805 | 0.00064 | 0.36834 |
| 5 | 288.203 | 210.138 | -0.8344 | -4.7878 | 0.00074 | 0.36834 |

| **day 6** |  |  |  |  |  |  |
| --- | --- | --- | --- | --- | --- | --- |
|  | **mzmed** | **rtmed** | **correlation** | **t-stat** | **p-value** | **FDR** |
| 1 | 360.195 | 323.359 | 0.95611 | 10.319 | 1.19E-06 | 0.00298 |
| 2 | 594.327 | 274.852 | 0.9436 | 9.0128 | 4.09E-06 | 0.0051 |
| 3 | 376.188 | 267.4 | 0.91673 | 7.2562 | 2.74E-05 | 0.02281 |
| 4 | 577.264 | 264.317 | 0.90204 | 6.6084 | 6.01E-05 | 0.03135 |
| 5 | 626.317 | 265.256 | 0.90119 | 6.5751 | 6.27E-05 | 0.03135 |
| 6 | 635.343 | 351.358 | 0.89556 | 6.365 | 8.19E-05 | 0.03412 |
| 7 | 427.257 | 269.461 | 0.86925 | 5.5601 | 0.00024 | 0.08592 |
| 8 | 440.296 | 351.024 | 0.85451 | 5.2021 | 0.0004 | 0.12497 |
| 9 | 495.158 | 618.963 | 0.839 | 4.8759 | 0.00065 | 0.17934 |
| 10 | 485.254 | 220.639 | 0.82585 | 4.6314 | 0.00093 | 0.22528 |
| 11 | 528.305 | 266.921 | 0.82188 | 4.5624 | 0.00104 | 0.22528 |
| 12 | 525.313 | 280.538 | 0.82031 | 4.5357 | 0.00108 | 0.22528 |
| 13 | 759.384 | 322.414 | 0.81546 | 4.4551 | 0.00123 | 0.23166 |
| 14 | 421.235 | 290.663 | 0.81317 | 4.4182 | 0.0013 | 0.23166 |
| 15 | 744.355 | 268.376 | 0.80698 | 4.321 | 0.00151 | 0.2394 |
| 16 | 759.384 | 324.099 | 0.80307 | 4.2618 | 0.00166 | 0.2394 |

| **day 10** |  |  |  |  |  |  |
| --- | --- | --- | --- | --- | --- | --- |
|  | **mzmed** | **rtmed** | **correlation** | **t-stat** | **p-value** | **FDR** |
| 1 | 177.696 | 607.113 | 0.99712 | 41.568 | 1.56E-12 | 3.89E-09 |
| 2 | 157.183 | 607.708 | 0.95856 | 10.64 | 8.98E-07 | 0.00112 |
| 3 | 784.864 | 239.207 | 0.95306 | 9.9536 | 1.66E-06 | 0.00138 |
| 4 | 546.771 | 221.806 | 0.91882 | 7.3616 | 2.42E-05 | 0.01512 |
| 5 | 764.359 | 287.504 | 0.90819 | 6.8617 | 4.40E-05 | 0.02197 |
| 6 | 801.407 | 311.235 | -0.8965 | -6.3988 | 7.84E-05 | 0.03267 |
| 7 | 178.197 | 567.67 | 0.88252 | 5.9342 | 0.00014 | 0.0515 |
| 8 | 541.344 | 339.467 | 0.86932 | 5.5621 | 0.00024 | 0.07498 |
| 9 | 594.326 | 277.274 | 0.8609 | 5.3509 | 0.00032 | 0.08975 |
| 10 | 440.296 | 366.233 | 0.83203 | 4.743 | 0.00079 | 0.17933 |
| 11 | 421.234 | 293.368 | 0.83169 | 4.7368 | 0.0008 | 0.17933 |
| 12 | 655.342 | 242.843 | 0.82825 | 4.674 | 0.00088 | 0.17933 |
| 13 | 539.283 | 236.399 | 0.82298 | 4.5814 | 0.00101 | 0.17933 |
| 14 | 721.389 | 286.436 | 0.81927 | 4.5182 | 0.00111 | 0.17933 |
| 15 | 159.149 | 211.955 | 0.81755 | 4.4895 | 0.00116 | 0.17933 |
| 16 | 539.283 | 235.222 | 0.81462 | 4.4416 | 0.00125 | 0.17933 |
| 17 | 539.283 | 238.834 | 0.81404 | 4.4321 | 0.00127 | 0.17933 |
| 18 | 539.283 | 237.6 | 0.81336 | 4.4212 | 0.00129 | 0.17933 |
| 19 | 769.385 | 234.523 | 0.81036 | 4.3734 | 0.00139 | 0.18303 |
| 20 | 558.252 | 214.599 | 0.808 | 4.3367 | 0.00147 | 0.18418 |
| 21 | 370.707 | 222.697 | 0.80375 | 4.2719 | 0.00163 | 0.1847 |
| 22 | 179.093 | 962.18 | 0.80222 | 4.2491 | 0.00169 | 0.1847 |
| 23 | 428.251 | 284.273 | 0.80092 | 4.2299 | 0.00174 | 0.1847 |
| 24 | 366.208 | 292.638 | 0.80019 | 4.2192 | 0.00177 | 0.1847 |

| **day 14** |  |  |  |  |  |  |
| --- | --- | --- | --- | --- | --- | --- |
|  | **mzmed** | **rtmed** | **correlation** | **t-stat** | **p-value** | **FDR** |
| 1 | 698.338 | 259.939 | 0.89051 | 6.1896 | 0.0001 | 0.11734 |
| 2 | 624.337 | 274.534 | 0.88544 | 6.0247 | 0.00013 | 0.11734 |
| 3 | 313.358 | 608.771 | 0.8831 | 5.952 | 0.00014 | 0.11734 |
| 4 | 769.385 | 234.589 | 0.85503 | 5.2139 | 0.00039 | 0.20944 |
| 5 | 639.67 | 301.207 | 0.84967 | 5.0954 | 0.00047 | 0.20944 |
| 6 | 189.087 | 239.072 | 0.84481 | 4.993 | 0.00054 | 0.20944 |
| 7 | 546.771 | 223.038 | 0.84225 | 4.9406 | 0.00059 | 0.20944 |
| 8 | 177.696 | 606.808 | 0.83062 | 4.7171 | 0.00082 | 0.25617 |
| 9 | 175.108 | 96.4332 | 0.82047 | 4.5384 | 0.00108 | 0.2831 |
| 10 | 187.145 | 273.373 | 0.81371 | 4.4269 | 0.00128 | 0.2831 |
| 11 | 456.217 | 310.451 | -0.81337 | -4.4214 | 0.00129 | 0.2831 |
| 12 | 511.288 | 295.187 | 0.8113 | 4.3884 | 0.00136 | 0.2831 |
| 13 | 187.144 | 268.596 | 0.80602 | 4.3064 | 0.00155 | 0.29717 |

| **day 21** |  |  |  |  |  |  |
| --- | --- | --- | --- | --- | --- | --- |
|  | **mzmed** | **rtmed** | **correlation** | **t-stat** | **p-value** | **FDR** |
| 1 | 651.347 | 271.765 | 0.94801 | 9.4204 | 2.74E-06 | 0.00354 |
| 2 | 178.197 | 575.459 | 0.94167 | 8.8485 | 4.82E-06 | 0.00354 |
| 3 | 546.771 | 221.56 | 0.9403 | 8.737 | 5.40E-06 | 0.00354 |
| 4 | 546.771 | 217.966 | 0.93796 | 8.5539 | 6.52E-06 | 0.00354 |
| 5 | 313.358 | 575.459 | 0.93691 | 8.4753 | 7.08E-06 | 0.00354 |
| 6 | 792.426 | 323.84 | 0.92549 | 7.7268 | 1.59E-05 | 0.00586 |
| 7 | 357.187 | 211.238 | 0.92505 | 7.7013 | 1.64E-05 | 0.00586 |
| 8 | 366.208 | 292.433 | 0.92242 | 7.5529 | 1.94E-05 | 0.00606 |
| 9 | 366.208 | 294.637 | 0.91106 | 6.9881 | 3.77E-05 | 0.01036 |
| 10 | 584.341 | 233.511 | 0.9093 | 6.9097 | 4.15E-05 | 0.01036 |
| 11 | 370.707 | 215.552 | 0.90532 | 6.7403 | 5.10E-05 | 0.01133 |
| 12 | 370.706 | 218.525 | 0.90405 | 6.6887 | 5.44E-05 | 0.01133 |
| 13 | 414.235 | 230.003 | 0.89976 | 6.52 | 6.72E-05 | 0.01282 |
| 14 | 472.258 | 210.827 | 0.89838 | 6.4681 | 7.18E-05 | 0.01282 |
| 15 | 404.214 | 216.292 | 0.89331 | 6.2854 | 9.08E-05 | 0.01513 |
| 16 | 177.696 | 575.459 | 0.88574 | 6.0341 | 0.00013 | 0.01972 |
| 17 | 482.736 | 258.093 | 0.88042 | 5.8713 | 0.00016 | 0.02308 |
| 18 | 666.868 | 325.247 | 0.87829 | 5.8089 | 0.00017 | 0.02372 |
| 19 | 415.239 | 230.004 | 0.87208 | 5.6354 | 0.00022 | 0.02851 |
| 20 | 420.367 | 608.643 | 0.86723 | 5.5078 | 0.00026 | 0.03236 |
| 21 | 866.446 | 293.533 | 0.86552 | 5.4644 | 0.00028 | 0.03275 |
| 22 | 666.868 | 327.654 | 0.86417 | 5.4308 | 0.00029 | 0.03278 |
| 23 | 419.866 | 608.643 | 0.86271 | 5.3948 | 0.0003 | 0.03299 |
| 24 | 573.289 | 263.65 | 0.85979 | 5.3244 | 0.00034 | 0.03356 |
| 25 | 667.369 | 327.654 | 0.85978 | 5.3241 | 0.00034 | 0.03356 |
| 26 | 865.443 | 293.694 | 0.85631 | 5.2433 | 0.00038 | 0.03624 |
| 27 | 536.309 | 322.285 | 0.85349 | 5.1792 | 0.00041 | 0.03732 |
| 28 | 624.337 | 274.678 | 0.85314 | 5.1715 | 0.00042 | 0.03732 |
| 29 | 177.696 | 565.249 | 0.84878 | 5.0764 | 0.00048 | 0.04118 |
| 30 | 584.341 | 226.305 | 0.84774 | 5.0541 | 0.0005 | 0.04118 |
| 31 | 346.209 | 249.758 | 0.84578 | 5.013 | 0.00053 | 0.04118 |
| 32 | 184.704 | 610.821 | 0.84577 | 5.0128 | 0.00053 | 0.04118 |
| 33 | 529.299 | 303.384 | 0.83892 | 4.8744 | 0.00065 | 0.04813 |
| 34 | 1033.53 | 303.368 | 0.83853 | 4.8667 | 0.00065 | 0.04813 |
| 35 | 293.176 | 231.506 | 0.83156 | 4.7343 | 0.0008 | 0.05624 |
| 36 | 661.304 | 292.75 | 0.83106 | 4.7251 | 0.00081 | 0.05624 |
| 37 | 511.288 | 294.465 | 0.82871 | 4.6824 | 0.00086 | 0.05838 |
| 38 | 626.294 | 237.509 | 0.8253 | 4.6216 | 0.00095 | 0.06162 |
| 39 | 278.166 | 226.194 | 0.82455 | 4.6086 | 0.00097 | 0.06162 |
| 40 | 293.176 | 233.329 | 0.82382 | 4.5959 | 0.00099 | 0.06162 |
| 41 | 855.5 | 405.788 | 0.8224 | 4.5713 | 0.00102 | 0.06222 |
| 42 | 292.674 | 233.329 | 0.82126 | 4.5517 | 0.00106 | 0.06222 |
| 43 | 159.149 | 207.645 | 0.82071 | 4.5424 | 0.00107 | 0.06222 |
| 44 | 565.263 | 277.22 | 0.81656 | 4.4732 | 0.00119 | 0.06767 |
| 45 | 430.23 | 230.907 | 0.81391 | 4.43 | 0.00127 | 0.07076 |
| 46 | 660.3 | 292.642 | 0.81296 | 4.4148 | 0.0013 | 0.07088 |
| 47 | 674.866 | 320.396 | 0.80956 | 4.361 | 0.00142 | 0.07406 |
| 48 | 471.257 | 243.08 | 0.80851 | 4.3446 | 0.00146 | 0.07406 |
| 49 | 558.288 | 253.137 | 0.80805 | 4.3375 | 0.00147 | 0.07406 |
| 50 | 157.183 | 607.093 | 0.80778 | 4.3333 | 0.00148 | 0.07406 |
| 51 | 657.303 | 222.762 | 0.80392 | 4.2745 | 0.00163 | 0.07943 |
| 52 | 284.658 | 93.9447 | 0.80264 | 4.2554 | 0.00168 | 0.07943 |
| 53 | 657.805 | 222.849 | 0.80173 | 4.2419 | 0.00171 | 0.07943 |
| 54 | 357.715 | 277.382 | 0.80161 | 4.2401 | 0.00172 | 0.07943 |

| **day 28** |  |  |  |  |  |  |
| --- | --- | --- | --- | --- | --- | --- |
|  | **mzmed** | **rtmed** | **correlation** | **t-stat** | **p-value** | **FDR** |
| 1 | 177.696 | 598.291 | 0.96672 | 11.95 | 3.04E-07 | 0.00074 |
| 2 | 157.183 | 608.274 | 0.96196 | 11.135 | 5.88E-07 | 0.00074 |
| 3 | 313.358 | 598.475 | 0.95552 | 10.245 | 1.27E-06 | 0.00106 |
| 4 | 157.183 | 621.917 | 0.90368 | 6.6737 | 5.54E-05 | 0.03463 |
| 5 | 177.696 | 625.82 | 0.88325 | 5.9565 | 0.00014 | 0.06998 |
| 6 | 283.311 | 602.376 | 0.873 | 5.6603 | 0.00021 | 0.08724 |
| 7 | 246.041 | 205.801 | 0.84376 | 4.9714 | 0.00056 | 0.17522 |
| 8 | 153.114 | 1015.12 | 0.84172 | 4.9299 | 0.0006 | 0.17522 |
| 9 | 638.346 | 382.777 | 0.83979 | 4.8915 | 0.00063 | 0.17522 |
| 10 | 339.374 | 611.451 | 0.83483 | 4.7954 | 0.00073 | 0.18208 |
| 11 | 157.183 | 606.755 | 0.82793 | 4.6683 | 0.00088 | 0.18916 |
| 12 | 170.19 | 605.205 | 0.82689 | 4.6498 | 0.00091 | 0.18916 |
| 13 | 309.327 | 839.029 | 0.80483 | 4.2882 | 0.00159 | 0.30577 |
