# Supplementary material for: Metabolic Profiling of Rhizobacteria Serratia plymuthica and Bacillus subtilis Revealed Intra- and Interspecific Differences and Elicitation of Plipastatins and Short Peptides Due to Co-cultivation
Source: Front Microbiol. 2021 May 31;12:685224. doi: 10.3389/fmicb.2021.685224 (PMC8200778; doi:10.3389/fmicb.2021.685224)
Supplement: Supplementary Table 1 — Lipopeptides produced by B. subtilis B2g. [file Data_Sheet_1.zip › Supplementary Tables/Table 6.DOCX]

**Supplementary table 6**| Time points of detection of differentially induced mass features (*m/z*) in *Serratia plymuthica* 4Rx13 interaction with *S. plymuthica* AS9 compared to mono-cultivated strains and medium control

| **day** | **1** | **3** | **6** | **10** | **14** | **21** | **28** |
| --- | --- | --- | --- | --- | --- | --- | --- |
| 1 |  |  |  |  |  |  | 153.1137 |
| 2 |  |  |  | 157.1826 |  | 157.1826 | 157.1827 |
| 3 |  |  |  |  |  |  | 157.1827 |
| 4 |  |  |  |  |  |  | 157.1827 |
| 5 |  |  |  | 159.1493 |  | 159.1492 |  |
| 6 |  |  |  |  |  |  | 170.1904 |
| 7 |  |  |  |  | 175.1078 |  |  |
| 8 |  |  |  | 177.6959 | 177.6959 | 177.6957 | 177.696 |
| 9 |  |  |  |  |  | 177.6958 | 177.696 |
| 10 |  |  |  | 178.1973 |  | 178.1973 |  |
| 11 |  |  |  | 179.0928 |  |  |  |
| 12 |  |  |  |  |  | 184.7036 |  |
| 13 |  |  |  |  | 187.1445 |  |  |
| 14 |  |  |  |  | 187.1445 |  |  |
| 15 |  |  |  |  | 189.0872 |  |  |
| 16 | 233.0638 |  |  |  |  |  |  |
| 17 |  |  |  |  |  |  | 246.0409 |
| 18 |  |  |  |  |  | 278.1663 |  |
| 19 |  |  |  |  |  |  | 283.3109 |
| 20 |  |  |  |  |  | 284.6583 |  |
| 21 |  | 288.2035 |  |  |  |  |  |
| 22 |  |  |  |  |  | 292.6742 |  |
| 23 |  |  |  |  |  | 293.1757 |  |
| 24 |  |  |  |  |  | 293.1757 |  |
| 25 |  |  |  |  |  |  | 309.3269 |
| 26 |  |  |  |  | 313.3577 | 313.3576 | 313.3579 |
| 27 |  |  |  |  |  |  | 339.3738 |
| 28 |  |  |  |  |  | 346.2086 |  |
| 29 |  |  |  |  |  | 357.1873 |  |
| 30 |  |  |  |  |  | 357.7152 |  |
| 31 | 359.2297 |  |  |  |  |  |  |
| 32 |  |  | 360.195 |  |  |  |  |
| 33 |  |  |  | 366.2083 |  | 366.2082 |  |
| 34 |  |  |  |  |  | 366.2084 |  |
| 35 |  |  |  | 370.7066 |  | 370.7064 |  |
| 36 |  |  |  |  |  | 370.7066 |  |
| 37 |  |  | 376.188 |  |  |  |  |
| 38 |  |  |  |  |  | 404.2142 |  |
| 39 |  |  |  |  |  | 414.2352 |  |
| 40 |  |  |  |  |  | 415.2385 |  |
| 41 |  |  |  |  |  | 419.8657 |  |
| 42 |  |  |  |  |  | 420.3674 |  |
| 43 |  |  | 421.2353 | 421.2345 |  |  |  |
| 44 |  | 427.2567 | 427.2568 |  |  |  |  |
| 45 |  |  |  | 428.2512 |  |  |  |
| 46 |  |  |  |  |  | 430.2302 |  |
| 47 |  |  | 440.2964 | 440.2958 |  |  |  |
| 48 |  |  |  |  | 456.2173 |  |  |
| 49 |  |  |  |  |  | 471.2568 |  |
| 50 |  |  |  |  |  | 472.2584 |  |
| 51 |  |  |  |  |  | 482.7356 |  |
| 52 |  |  | 485.2543 |  |  |  |  |
| 53 |  |  | 495.1581 |  |  |  |  |
| 54 |  |  |  |  | 511.2885 | 511.2884 |  |
| 55 |  | 525.3135 | 525.3135 |  |  |  |  |
| 56 |  |  | 528.3047 |  |  |  |  |
| 57 |  |  |  |  |  | 529.2989 |  |
| 58 |  |  |  |  |  | 536.309 |  |
| 59 |  |  |  | 539.2831 |  |  |  |
| 60 |  |  |  | 539.2831 |  |  |  |
| 61 |  |  |  | 539.2831 |  |  |  |
| 62 |  |  |  | 539.2832 |  |  |  |
| 63 |  | 541.3441 |  | 541.3435 |  |  |  |
| 64 |  |  |  | 546.7705 | 546.7706 | 546.7706 |  |
| 65 |  |  |  |  |  | 546.7707 |  |
| 66 |  |  |  | 558.2522 |  | 558.2884 |  |
| 67 |  | 563.3235 |  |  |  |  |  |
| 68 |  |  |  |  |  | 565.263 |  |
| 69 |  |  |  |  |  | 573.289 |  |
| 70 |  |  | 577.2638 |  |  |  |  |
| 71 |  |  |  |  |  | 584.341 |  |
| 72 |  |  |  |  |  | 584.3412 |  |
| 73 |  |  | 594.3269 | 594.3257 |  |  |  |
| 74 |  |  |  |  | 624.3366 | 624.3366 |  |
| 75 |  |  |  |  |  | 626.2942 |  |
| 76 |  |  | 626.3169 |  |  |  |  |
| 77 |  |  | 635.3425 |  |  |  |  |
| 78 |  |  |  |  |  |  | 638.3459 |
| 79 |  |  |  |  | 639.67 |  |  |
| 80 |  |  |  |  |  | 651.3473 |  |
| 81 |  |  |  | 655.3415 |  |  |  |
| 82 |  |  |  |  |  | 657.3032 |  |
| 83 |  |  |  |  |  | 657.8046 |  |
| 84 |  |  |  |  |  | 660.3001 |  |
| 85 |  |  |  |  |  | 661.3036 |  |
| 86 |  |  |  |  |  | 666.868 |  |
| 87 |  |  |  |  |  | 666.8681 |  |
| 88 |  |  |  |  |  | 667.3694 |  |
| 89 |  |  |  |  |  | 674.8662 |  |
| 90 |  |  |  |  | 698.3379 |  |  |
| 91 |  |  |  | 721.3894 |  |  |  |
| 92 |  |  | 744.3552 |  |  |  |  |
| 93 |  |  | 759.3837 |  |  |  |  |
| 94 |  |  | 759.3838 |  |  |  |  |
| 95 |  |  |  | 764.3587 |  |  |  |
| 96 |  |  |  | 769.3847 | 769.3849 |  |  |
| 97 |  |  |  | 784.8637 |  |  |  |
| 98 |  |  |  |  |  | 792.4258 |  |
| 99 |  |  |  | 801.4066 |  |  |  |
| 100 |  |  |  |  |  | 855.4999 |  |
| 101 |  |  |  |  |  | 865.4427 |  |
| 102 |  |  |  |  |  | 866.4458 |  |
| 103 |  |  |  |  |  | 1033.533 |  |
